# Molecular Structure–Tribological Performance Relationship in Tungsten-Based Polyoxometalate Ionic Liquid Lubricant Additives

**DOI:** 10.1021/acsomega.5c12084

**Published:** 2026-08-13

**Authors:** Maria Luisa Casasin-Garcia, Scott Mitchell, Nuria Espallargas

**Affiliations:** † Norwegian Tribology Center, Department of Mechanical and Industrial Engineering, Norwegian University of Science and Technology (NTNU) 7491, Trondheim, Norway; ‡ Instituto de Nanociencia y Materiales de Aragón (INMA-CSIC), 16379 Consejo Superior de Investigaciones Científicas, Universidad de Zaragoza 50009, Zaragoza, Spain

## Abstract

Polyoxometalate ionic liquids (POM-ILs) represent a class of multifunctional materials combining the structural tunability of polyoxometalates (POMs) with the versatile properties of ionic liquids (ILs). In this work, a series of POM-ILs were designed, synthesized, and evaluated as lubricant additives and as plain lubricants to demonstrate the relationship between their molecular structures and tribological performance. The influence of both the cationic moiety (ammonium vs phosphonium) and the anion structure (SiW_11_ vs SiW_9_) was investigated on two metallic substrates, AISI 316L stainless steel and AISI 52100 bearing steel, under boundary lubrication conditions. Comprehensive characterization combining friction and wear measurements, QCM-D adsorption analysis, and surface and subsurface chemical and structural analysis (XPS, SEM, and FIB-SEM) revealed that the POM-IL molecular structure critically influences friction and wear behavior. Increasing the lacunarity and negative charge density of the POM enhanced antiwear performance and reduced friction, likely through the formation of viscoelastic layers that promote surface separation. In addition, the base lubricant (PAO8) was found to be detrimental to the POM-IL surface interactions, reducing their efficiency as additives compared to their plain form. The results obtained in this work demonstrate that POM-ILs offer an effective alternative to conventional antiwear additives such as ZDDP, combining high molecular designability, strong substrate activity, and enhanced antiwear and friction-reducing behavior. These insights establish a structure-to-function relationship and highlight the potential of molecularly engineered POM-ILs for next-generation lubrication systems.

## Graphical abstract

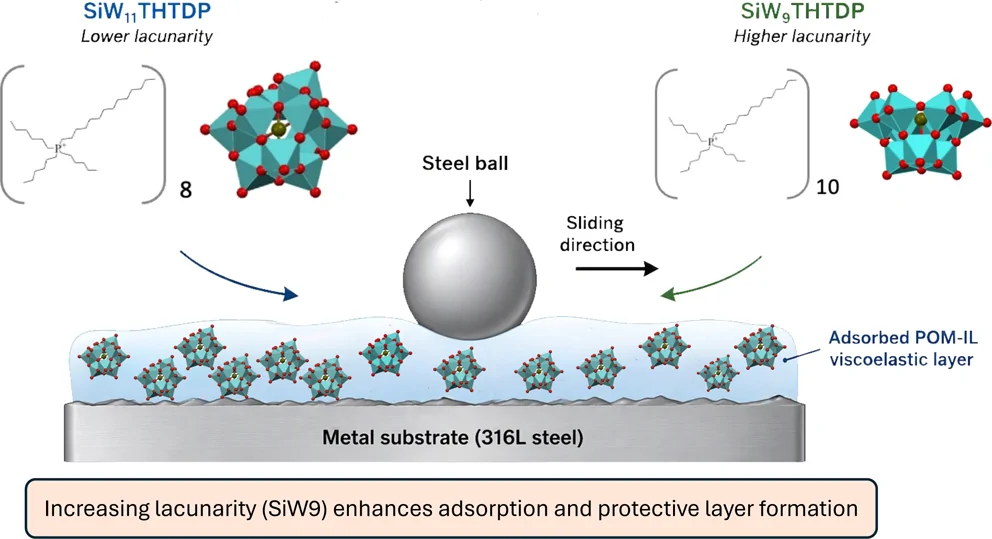

## 1 Introduction

Polyoxometalate-ionic liquids (POM-ILs) are compounds based on the ionic interaction of metal-oxide anion clusters and ionic liquid (IL) cations. The combination of both moieties is a straightforward process, rooted in the electrostatic attraction between ions of opposing charge. Additionally, each separate component can be engineered precisely in terms of molecular and 3D structure, polarity, symmetry, and nucleophilicity, among others. A well-designed POM-IL compound can prompt an optimal performance in a wide array of fields, with catalysis being a particularly prominent example, where significant benefits have already been demonstrated.^1–4^

In a tribological context, POM-ILs as lubricant additives offer combined properties that have the potential to surpass those of their individual components. The POM moiety provides several structural advantages as a lubricant additive. Key elements such as sulfur (S), phosphorus (P), oxygen (O), and molybdenum (Mo) can contribute to the POM’s role as lubricant additives.^5,6^ The high negative charge density of POMs enhances their interaction with metal surfaces, leading to the potential formation of protective films that could reduce friction and wear.^7^ The IL moiety of POM-ILs acts as a compatibility enhancer with the base lubricants, improving solubility and dispersion of the POMs within the base lubricant. This compatibility is crucial for ensuring the uniform dispersion of the POMs, thereby maintaining consistent lubrication performance. Furthermore, the low volatility of ILs and the thermal stability of POMs minimize evaporation losses, which is particularly advantageous in high-temperature environments. This reduces the frequency of lubricant replenishment, improving overall efficiency. Consequently, POM-ILs exhibit enhanced thermal stability, reduced friction and wear, improved solubility in base lubricants, and superior longevity, making them a promising solution for advanced lubrication technologies.

Previous research work with POM-ILs in a tribological context has shown their potential as friction-reducing and antiwear (AW) lubricant additives on stainless steel.^7^ From that work, one POM-IL structure (SiW_11_O_39_THTDA) used as an additive in a low-viscosity polyalphaolefin (PAO8) showed successful results. The current work is built with that study as a basis, with the aim to rationally design new POM-IL structures that would maintain the compatibility with an environmentally acceptable base lubricant (PAO8) while targeting the desired AW and friction-reducing multifunctionality. The modifications of both the anion and the cation in POM-ILs have been undertaken to achieve a deeper understanding of the structure-to-performance relationship of this promising new class of lubricant additives.

The first modification from the initial POM-IL (SiW_11_O_39_THTDA) involves swapping the ammonium-based cation (THTDA) for a phosphonium one (THTDP). This first structural change aims to improve the environmental compatibility of the compound as well as introduce a phosphorus atom into the structure, which would be more active in a tribological context. The influence of negative charge on the strength of adsorption, subsequent substrate interactions, and tribological performance has also been explored by modifying the POM structure. The lacunarity of a POM refers to the number of void spaces present in its molecular structure relative to its plenary form, as illustrated in Figure 1. To study the effect of negative charge (or nucleophilic character) on the tribological performance, the removal of two tetrahedral units (WO_4_) from the original structure (the monolaculary SiW_11_O_39_) was carried out. This change increases the lacunarity and the charge density in the POM moiety and could potentially improve the affinity for the positively charged metal surfaces under tribological conditions.^8^

**Figure 1:**
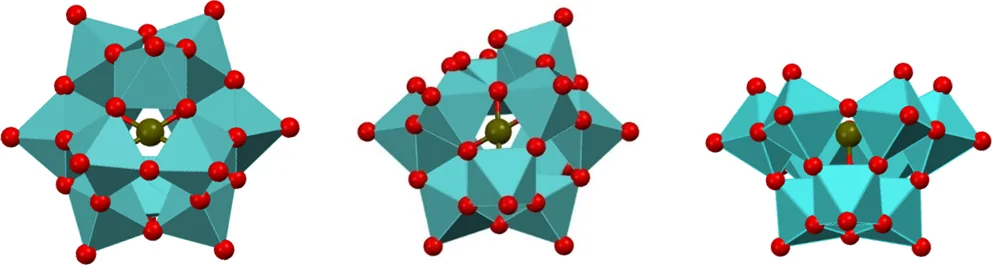
Plenary (left) and mono (center) and tri (right) lacunary Keggin structures corresponding to the formulas: [XM_12_O_40_]^n–^, [XM_11_O_39_]^(n+4)‑^, and [XM_9_O_34_] ^(n+6)‑^, respectively.

The present study investigates the effect of POM-ILs’ chemical structure when used as lubricant additives in a synthetic low-viscosity polyalphaolefin on two different metallic substrates: AISI 52100 bearing steel and AISI 316L stainless steel. These two steels differ in chemical composition, mechanical properties, and performance under similar conditions, leading to distinct applications. 52100 is a high-carbon steel known for its increased wear resistance and fatigue strength, making it ideal for use in rolling bearings. In contrast, 316L has a high chromium and nickel content, providing enhanced corrosion protection but with a softer surface, making it more prone to wear and a shorter lifespan. This material is commonly used in environments characterized by the presence of moisture.

To achieve the objectives of the study, the POMs and POM-ILs used in this work were synthesized and characterized through Fourier transform infrared spectroscopy (FTIR) and thermogravimetric analysis (TGA). A primary zinc dialkyldithiophosphate (ZDDP), which is a widely used antiwear additive, was used for comparison. The additives were tested under two conditions:^1^ blended in polyalphaolefin 8 (PAO8) and studied as additives, and^2^ plain for their study as base lubricants. Subsequent friction and wear tests were then carried out. These tests included measuring the coefficient of friction (CoF) over time, measuring the wear volume with an infinite focus microscope (IFM), and imaging the wear scars with a scanning electron microscope (SEM). Then, surface adsorption analysis was performed on two metallic sensors (stainless steel and iron-coated) by means of a quartz crystal microbalance equipped with dissipation monitoring (QCM-D). Finally, surface chemical analysis was performed with X-ray photoelectron spectroscopy (XPS) and scanning electron microscopy with a focused ion beam (SEM-FIB) for studying the chemical interaction of these structures with the metallic substrates.

## 2 Experimental Section

### 2.1 Materials

Table 1 shows the structure of the synthesized POM-ILs along with some selected properties. They are all silicotungstate Keggin polyoxometalates of varying lacunarity joined by either P- or N-based cations containing long aliphatic chains. A well-known antiwear additive, zinc dialkyldithiophosphate (ZDDP), purchased from Lanxess, was used as a comparison. The base lubricant used for the preparation of the lubricant blends consisted of polyalphaolefin 8 (PAO8, where 8 corresponds to its kinematic viscosity in cSt at 100 °C) provided from Chevron Phillips. This base lubricant is a hydrocarbon chain blend formed through the oligomerization of linear α-olefins, which results in the formation of varying chain lengths and branching. PAO8 is a low-viscosity base lubricant, and it is considered to be environmentally acceptable.^9^ PAO8 and ZDDP were used as received without further modification.

**Table 1:**
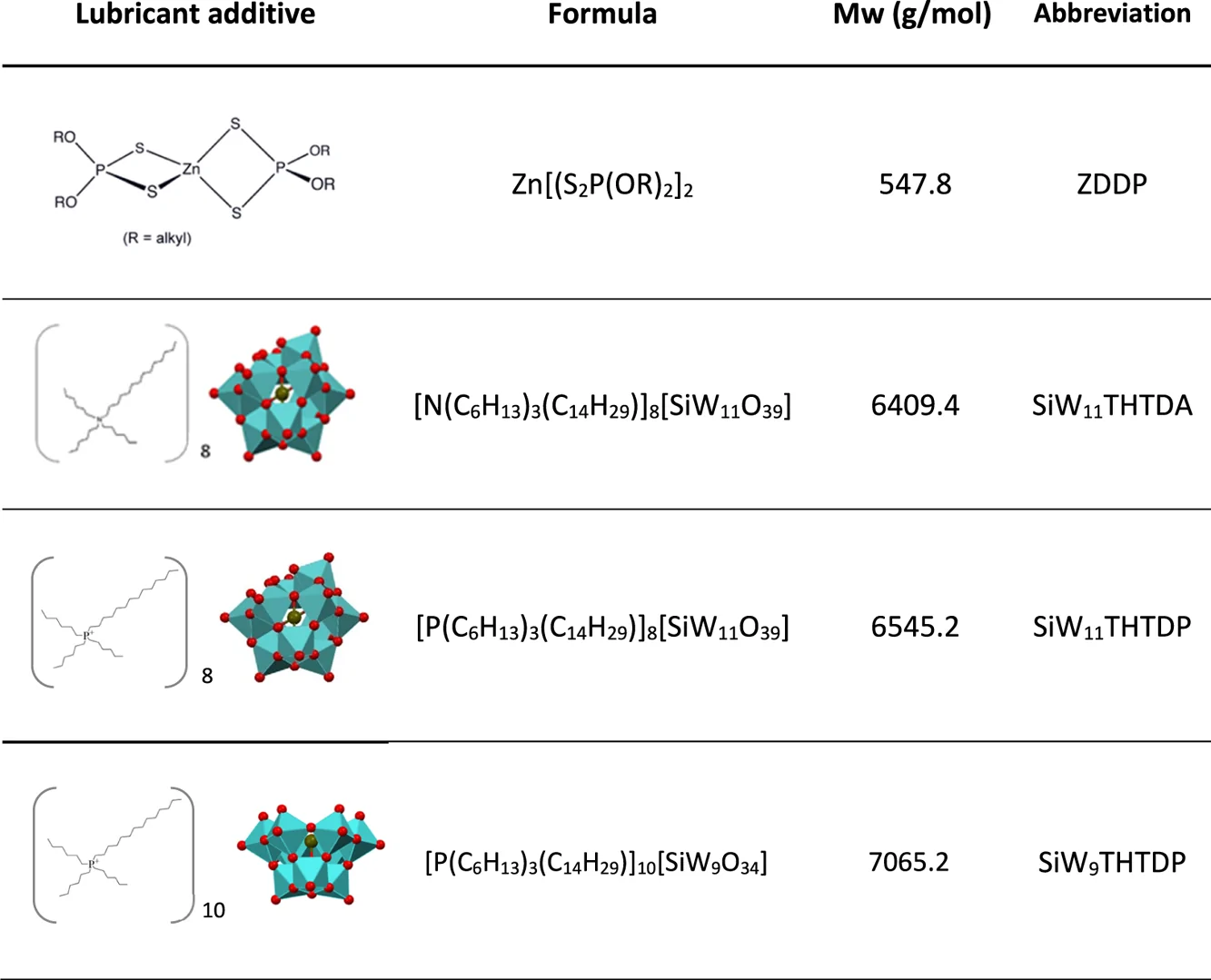
Structure and Properties of Synthesized POM-ILs and ZDDP

As metallic substrates for the tribological testing, AISI 316L stainless steel (316L) and annealed AISI 52100 bearing steel (52100) were used. For further information about the metals’ microstructures and properties, the reader is directed to our previous paper.^10^ The cylindrical substrates were cut into 6 mm thick and 30 mm diameter discs by the manufacturer. Prior to testing, a polishing process was followed according to Struers specifications^11^ to ensure an adequate surface roughness (Ra ∼ 0.090 ± 0.003 μm). After polishing, the discs were thoroughly cleaned in an ultrasonic bath with a distilled water–ethanol mixture, rinsed with distilled water, and dried with pressurized air. The counterpart material (balls) for the ball-on-disc testing consisted of 6 mm diameter Al_2_O_3_ balls purchased from Precision Ball and Gauge Co., Ltd., and used without further modification.

### 2.2 POM-IL Synthesis and Characterization

The POM-IL structures were synthesized in two steps. First, the POM moieties were prepared from precursors purchased from Sigma-Aldrich or Acros Organics at reagent grade and used without further purification. These were then mixed with the IL (purchased from Sigma-Aldrich), thus obtaining the POM-ILs. The synthesis of the POM salts (Na_10_[SiW_9_O_34_] and K_8_[SiW_11_O_39_]) was carried out following procedures available in the literature,^12^ with small adjustments. Following the synthesis, FTIR and TGA analysis were performed for characterization. An FTIR spectrometer (PerkinElmer, USA) equipped with an ATR mode was used to carry out all measurements. The Spectrum 10 software was used in the 4000–350 cm^–1^ region with a resolution of 2 cm^–1^. TGA analyses were carried out in air, between the temperatures of 25 and 900 °C, on a TGA Q5000 (Waters, TA Instruments, Deutschland).^13,14^

#### 2.2.1 Synthesis of K_8_[SiW_11_O_39_]

Sodium metasilicate (0.50 g, 4.09 mmol) was dissolved in 10 mL of distilled water at room temperature. This solution was filtered, and the solid was discarded. In another 100 mL beaker, sodium tungstate (8.26 g, 25.18 mmol) was dissolved in 3 mL of distilled water at 100 °C. A precipitate will appear in the tungstate solution; to dissolve it, 8.25 mL of 4 M HCl_(aq)_ solution was added dropwise over 5 min with vigorous stirring to the boiling tungstate solution. To the latter, the metasilicate solution was added, quickly followed by the addition of 2.50 mL of 4 M HCl_(aq)_ solution. The resulting solution remained under boiling for 1 h. After cooling down to room temperature, the solution was filtered and the solid discarded. Potassium chloride (6.80 g, 91.2 mmol) was added to the filtered solution under stirring. The resulting white precipitate was collected on a funnel with a medium porosity sintered disc, washed with two 20 mL fractions of 1 M KCl_(aq)_, 50 mL of cold water, and finally dried in air (yield: 5.10 g, 1.58 mmol, 69.7%). FT-IR (cm^–1^): 3420 (b), 2364 (w), 2037 (m), 1624 (m), 995 (s), 917 (s), 888 (vs), 793 (vs), 512 (s), 480 (s).

#### 2.2.2 Synthesis of Na_10_[SiW_9_O_34_]

Sodium tungstate (30.33 g, 91.95 mmol) and sodium metasilicate (1.83 g, 14.96 mmol) were dissolved in 100 mL of distilled water at 80–100 °C with a stirring magnet. Upon dissolution of the solids, 22 mL of 6 M HCl_(aq)_ was added dropwise during a 30 min interval. This solution was then filtered, and the solid was discarded. Simultaneously, sodium carbonate (8.30 g, 78.31 mmol) was dissolved in 30 mL of distilled water at 40 °C. Once the carbonate was dissolved, it was added slowly to the tungstate and metasilicate solution. The resulting solution was kept under stirring for 1h, during which a white precipitate appeared. After 1 h, the solution was filtered, and the liquid was discarded. This solid was later recrystallized with 4 M NaCl_(aq)_ and dried in air (yield: 6.43 g, 2.41 mmol, 21% yield). FT-IR (cm^–1^): 3182 (b), 1640 (w), 1091 (w), 980 (m), 929 (m), 839 (vs), 799 (vs), 675 (s), 553 (m), 524 (m)

#### 2.2.3 Synthesis of SiW_11_[THTDA]_8_, SiW_11_[THTDP]_8_, and SiW_9_[THTDP]_10_

The procedure for POM-IL preparation was similar for all cases, except for the molar ratio (1:8 and 1:10 for SiW_11_ and SiW_9_, respectively) and the cation choice (ammonium or phosphonium). After the appropriate ratio and reagents were chosen, the procedure started by dissolving the POM salt (around 1 mmol) in 50 mL of distilled water at 50 °C. Simultaneously, the cation was dissolved in 80 mL of toluene. The POM solution was slowly added to the IL-toluene and left under stirring for 30 min at room temperature. After 30 min, the solution was transferred to a separatory funnel, and two phases were observed, where the top was the POM-IL containing toluene and the bottom was water, to be discarded. The toluene fraction was then decanted to a round-bottom flask and subjected to evaporation of the solvent under pressure (rotavapor). The resulting products were highly viscous liquids of dark yellow/orange and pale yellow colors for THTDA and THTDP, respectively.

FTIR (cm^–1^): SiW_11_[THTDA]_8_: 2923 (s), 2853 (m), 1466 (m), 1378 (w), 946 (m), 919 (m), 886 (vs), 799 (s), 775 (s), 729 (s), 529 (w). TGA: 43% (calculated 41.7%) SiW_11_[THTDP]_8_: 2922 (s), 2853 (m), 1465 (w), 986 (w), 942 (w), 882 (vs), 774 (s), 727 (vs), 523 (w). TGA: 46% (calculated 40.8%) SiW_9_[THTDP]_10_: 2923 (s), 2854 (m), 1465 (w), 980 (w), 926 (w), 870 (s), 796 (w), 727 (vs), 694 (m), 513 (w). TGA: 38% (calculated 31.5%).

### 2.3 Lubricant Blends

The lubricant blends were prepared by mixing 1 wt % of additive with 99 wt % PAO8. The blends were mixed with a magnetic stirrer at 600 rpm for 6 h at 40 °C and were then subjected to an ultrasonic bath for approximately 1 h. The blends were allowed to cool to room temperature before testing. All additives on PAO8 formed emulsions, similar to what was described in previous work,^7^ and thus were considered compatible to proceed with tribological testing. Plain POM-ILs and ZDDP were also tested unblended for comparison. However, SiW_11_THTDA was not tested plain due to the small quantity available.

### 2.4 Tribological Testing

The tribological experiments were conducted using a ball-on-disc tribometer (TriboCorr, Resmat Corporation, Canada) under boundary lubrication conditions, as determined by the Hamrock-Dawson eq (13). The different steel samples underwent reciprocal sliding motion against a 6 mm diameter Al_2_O_3_ ball at a 20 N normal load, corresponding to a maximum initial Hertzian contact pressure of 2.05 GPa. The tests were carried out with 1 mL of lubricant at room temperature, with a sliding frequency of 1 Hz and a 10 mm stroke length, resulting in a linear velocity of 0.02 m/s. Each test lasted for 5000 cycles (20 mm per cycle), amounting to a total sliding distance of 100 m. To ensure repeatability, all tests were repeated at least twice.

### 2.5 Adsorption Studies

The adsorption behavior of the POM-ILs and ZDDP on the metal substrates was studied with a QCM-D from Biolin Scientific AB (Sweden). This instrument was used to study how strong the additives’ affinity was for the different substrates in the base lubricant and provide insight into the viscoelastic properties of the film formed. The Sauerbrey equation establishes the relationship between changes in frequency (Δ*f*) of the quartz crystal disc and mass added (Δ*m*) onto the sensor. It is important to consider that this equation, however, assumes the formation of rigid and thin layers.

$$\Delta f=-\frac{2f_{0}^{2}}{A\rho_{q}}\Delta m\;Sauerbrey\;equation$$

where *f*_0_ describes the fundamental quartz crystal frequency under vacuum, *A* is the crystal surface area, and ρ_q_ is the quartz density.

The sensors used during this work were either stainless steel or iron coated for simulating 316L and 52100 substrates, respectively, and were purchased from RenLux Crystal (China). Prior to the adsorption studies, the sensors were thoroughly cleaned with an ultrasonic bath and UV–ozone cleaner. Then, the experiments consisted of pumping 50 mL/min of PAO8, PAO8 + additive, and finally PAO8 for rinsing. Throughout the process, both the frequency and dissipation were monitored with time. The plain POM-ILs and ZDDP could not undergo adsorption studies given the QCM instrument limitations for viscosity. All tests were repeated at least twice to verify the repeatability of the results.

### 2.6 Materials Characterization

The wear scar of the tested steel samples was measured using an Alicona Infinite Focus 3D optical microscope (Bruker, US). Three different measurements were collected alongside the wear track of each tested steel sample and analyzed with MountainsMap software (Digital Surf, France). which provided the wear area at the specific points. These were then multiplied by the stroke length and averaged for each tested sample, making the data more accurate. This average wear volume was then used for the calculation of the specific wear rate (SWR) according to Archard’s eq (14).

$$SWR\left(\frac{{mm}^{3}}{Nm}\right)=\frac{Wear\;volume}{Normalload\cdot Slidingdistance}Archard’sequation$$

In addition to the calculation of the SWR from the volume of the wear track, insights into the wear mechanism were obtained through the calculation of the degree of material loss (β), defined by the following equation

$$\beta =\frac{A_{Track}-A_{Ridges}}{A_{Track}}\cdot 100$$

The morphology of the wear tracks was studied with an FEI Quanta 650 FEG (Thermo Fisher Scientific, US) SEM using an Everhart–Thornley detector (ETD). The top view of the wear tracks was used to compare the effects of the different chemistries on the two metal substrates and to carry out visual examination of the extent of wear. For this purpose, different magnifications of the wear tracks were measured.

The cross sections of the tested discs were prepared and imaged using a dual-beam SEM-FIB (FEI Helios G4 UX Nanolab DualBeam), equipped with an In-Chamber secondary Electron detector (ICE). This setup enabled high-resolution imaging to evaluate the effect of the lubricants and additives on the subsurface microstructures. For each tested disc, a single cross section was prepared at the central region of the wear track. Prior to cross-section preparation to milling, two protective layers were deposited on the substrate surface: a carbon electron deposition and a carbon ion deposition. The milling process then created a cross section of approximately 36 μm depth, which was then imaged at high resolution with the electron beam. Through this process, detailed analysis of microstructural changes, grain morphologies, and early indications of tribofilm formation was achieved.

For a more detailed understanding of the chemical changes in the substrates, XPS was performed using a Kratos AXIS Supra spectrometer equipped with a monochromatic Al_Kα_ X-ray source (120 W, 8 mA/15 kV). Survey scans were performed with a pass energy of 160 eV and a step of 1 eV over an analysis area of 700 × 300 μm. High-resolution scans of selected elements were also performed with a pass energy of 20 and 0.1 eV step. In certain cases, material etching was carried out using a 5000 eV argon ion beam to obtain elemental depth profiles. The binding energies of elements of interests were calibrated using the C 1s reference peak at 284.6 eV. All XPS data were analyzed using CasaXPS software.

## 3 Results

### 3.1 Friction Results

The friction results have been divided into two sections, one treating the POM-ILs and ZDDP as additives in PAO8, while in the second, they have been used as plain compounds. In the latter case, SiW_11_THTDA was not included because of material scarcity for such testing.

#### 3.1.1 Friction Results of POM-ILs and ZDDP as Additives in PAO8

Figure 2a shows the friction evolution of plain PAO8 and all the additive-containing blends tested with 316L. PAO8 presented a long run-in period of around 45 m, almost half of the entire test length, which later stabilized at a coefficient of friction (COF) value of 0.1621. All additives decreased both the duration of the running-in and the stabilized COF. ZDDP showed the lowest length of the running-in period, around 8 m, quickly followed by the phosphonium POM-ILs, at around 10 m. SiW_11_THTDA showed a longer running-in, of around 16 m. Additionally, the averaged COF after the running-in for the additives has the following trend, from highest to lowest: PAO8-ZDDP (0.1228) > PAO8-SiW_11_THTDA (0.1218) > PAO8-SiW_11_THTDP (0.1160) > PAO8-SiW_9_THTDP (0.1134).

**Figure 2:**
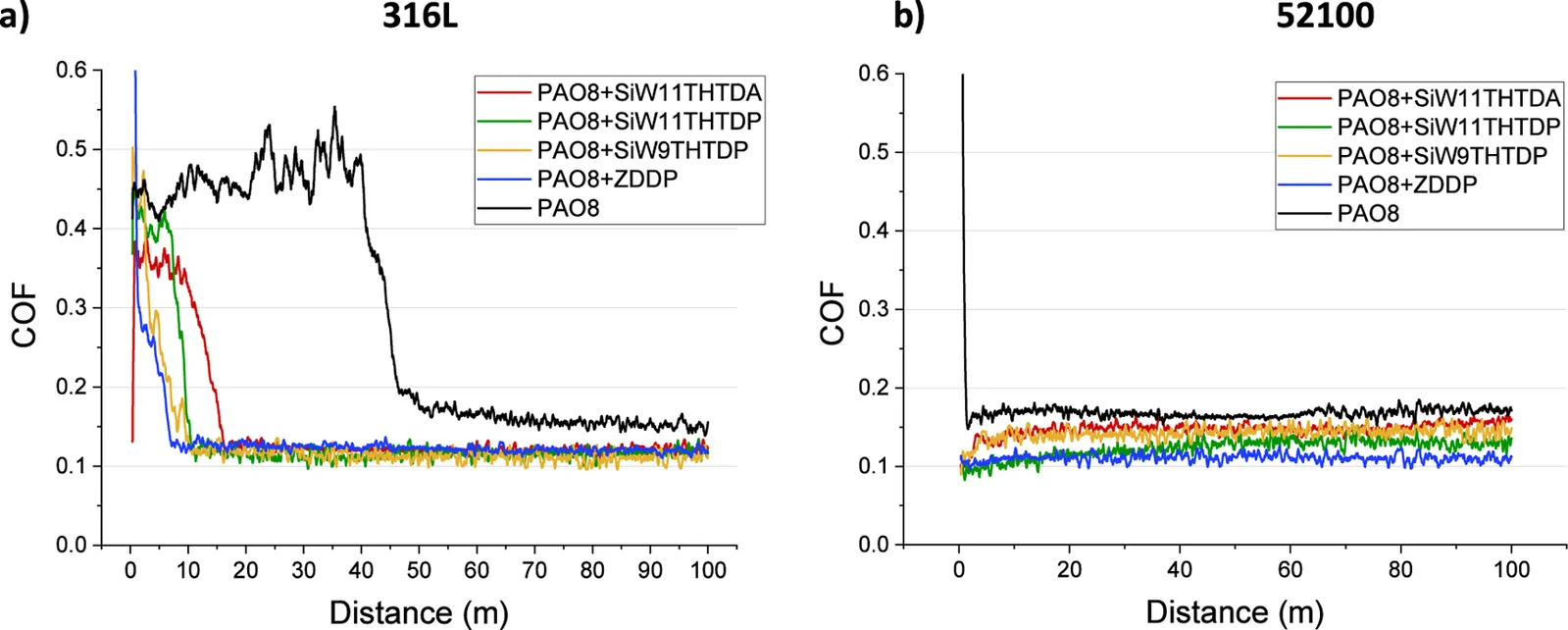
Friction evolution of the different lubricant additives in PAO8 in 316L (a) and 52100 (b).

In Figure 2b, the friction evolution is shown for the tests in 52100 bearing steel. The differences between lubricant blends containing additives and the plain base fluid were at a first glance less significant, which is mainly due to the absence of a running-in period for all blends. Plain PAO8 still presents the highest COF (0.1677) very similar to the same blend in 316L, followed by the POM-ILs: PAO8-SiW_11_THTDA (0.1473) > PAO8-SiW_9_THTDP (0.1421) > PAO8-SiW_11_THTDP (0.1233), and finally by PAO8-ZDDP (0.1107). Interestingly, the COF order is different for 52100 than for 316L, with ZDDP ranking the opposite between the two metals. Further information on the average COF and repeatability can be found in Table S1 in the Supporting Information.

#### 3.1.2 Friction Results of Plain POM-ILs and ZDDP

To better understand the metal-additive interaction, plain SiW_11_THTD and SiW_9_THTDP were tested unblended along with ZDDP for comparison. As shown in Figure 3a, the absence of a running-in period is evident for 316L, in contrast to the behavior observed when blending the POM-ILs and ZDDP in PAO8 (Figure 2a). Additionally, both POM-ILs exhibited the lowest COF throughout the test, achieving lower values than those when formulated in PAO8. In contrast, ZDDP maintains COF values similar to those observed for PAO8.

**Figure 3:**
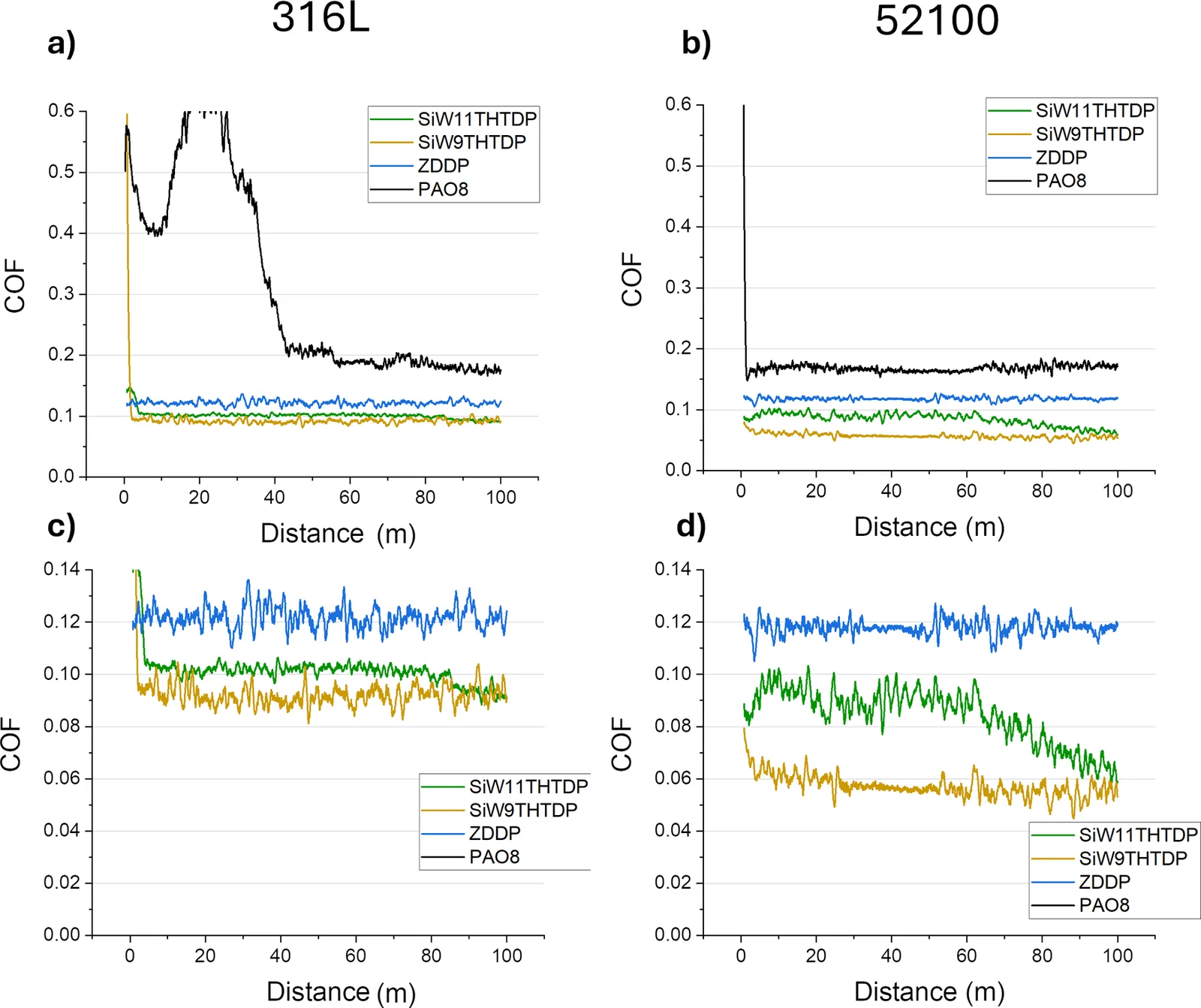
Friction evolution of two POM-ILs, ZDDP, and PAO8 lubricants in 316L (a, c) and 52100 (b, d).

Figure 3b presents the COF evolution on the 52100 substrate. The observed trends differ significantly from the behavior of the same compounds blended in PAO8 on the same substrate. In the plain case, ZDDP exhibited the highest COF throughout the test, by a considerable margin, whereas both POM-ILs demonstrated the lowest COF of all tests, with average values of around 0.06.

### 3.2 Adsorption of Additives Blended in PAO8 on the Metal Substrates

QCM-D has a recommended viscosity range for the fluids being analyzed, outside of which the results become suboptimal. The viscosities of the plain POM-ILs and ZDDP were well above this range, making it impossible to obtain adsorption data for these compounds alone. However, when prepared as additives in PAO8, all compounds were successfully measured, allowing for comparative analysis of their interactions with the metal substrates. The SiW_11_THTDA-PAO8 blend was particularly challenging to measure with QCM-D, as it interfered with the acquisition of complete experimental data. As a result, this blend was diluted to reduce viscosity and achieve a successful measurement. Consequently, the frequency drop and dissipation increase were less pronounced than for the other blends. Despite these limitations, the comparisons and observations from Figure 4 still provide valuable results. The QCM-D experiments followed the evolution of both frequency (Δ*f*) and dissipation (Δ*D*), both of which experienced changes upon fluid transitions.

**Figure 4:**
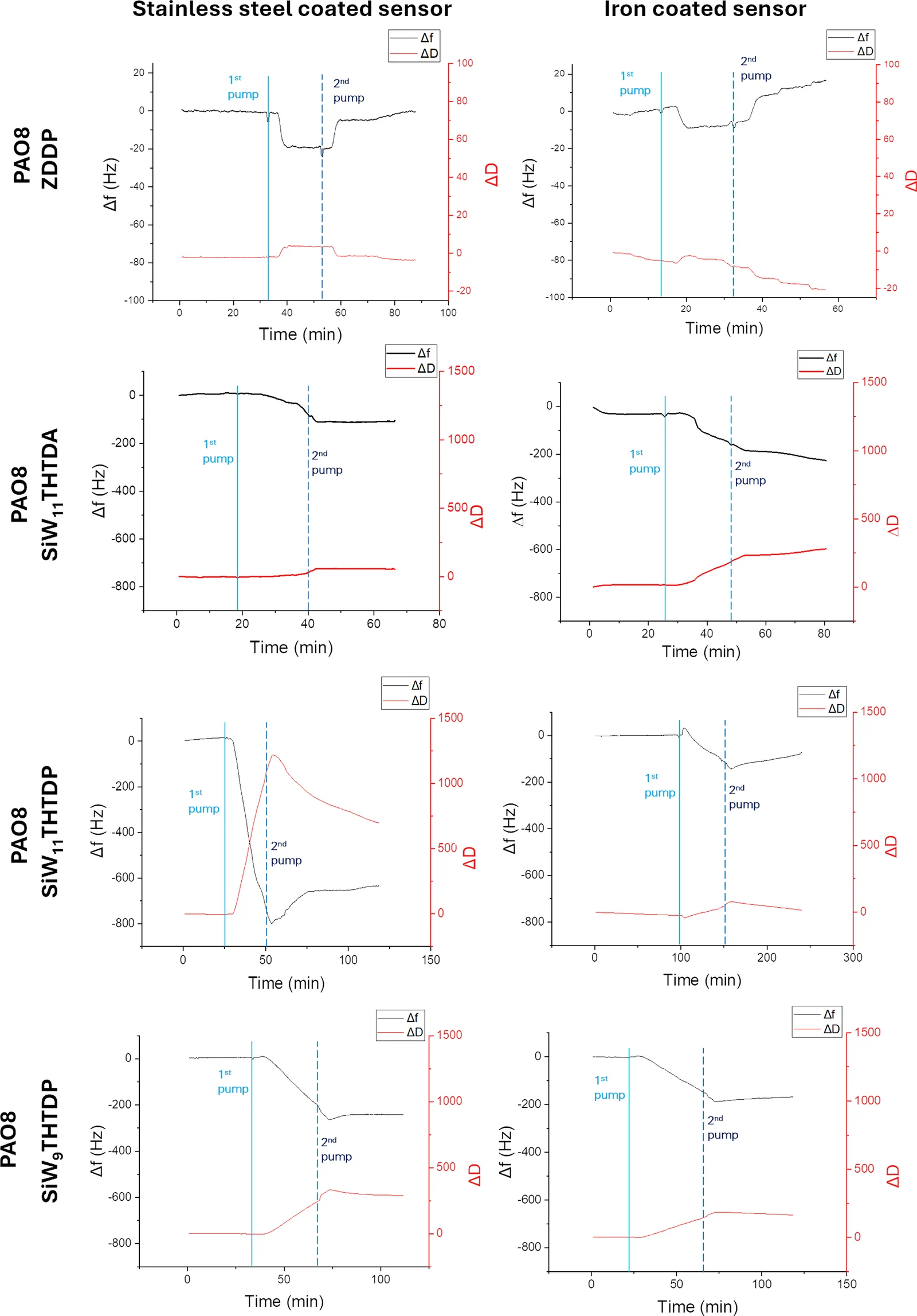
Adsorption results, in the form of frequency (Δ*f*) and dissipation­(Δ*D*) evolution over time for the tested blends of additives in PAO8 on stainless steel and iron-coated sensors. Two vertical lines indicate when the first (additive addition) and second (rinsing with base fluid) pump were carried out.

The first clear observation from Figure 4 is the distinct behavior of ZDDP compared to the POM-ILs. While ZDDP showed only minimal Δ*f* and Δ*D*, all POM-ILs consistently exhibited a large frequency drop accompanied by a significant increase in dissipation. Moreover, ZDDP showed a fast restoration of initial frequency values after rinsing with PAO8, suggesting a weaker, physisorption-driven interaction with both metal substrates. In contrast, all the POM-ILs showed a strong frequency drop and dissipation increase, indicative of a stronger affinity for both metallic substrates. This was further reinforced after the rinsing step, where frequency and dissipation failed to return to initial values, suggesting that the additive–substrate interaction leaned more toward strong chemisorption rather than physisorption.

Differences between the individual POM-ILs and each substrate are worth looking further into. First, SiW_11_THTDA required dilution for accurate monitoring, resulting in a less pronounced frequency drop and dissipation rise compared to the rest of the POM-ILs. However, this is expected considering the relationship between Δ*f* and Δ*m*. Despite that, SiW_11_THTDA demonstrated affinity for both substrates to a similar extent. SiW_11_THTDP showed much stronger affinity toward 316L than for 52100, which was clear by the largest frequency drop of all the blends, around 800 Hz, compared to around 200 Hz for 52100. This was accompanied by a similar effect on dissipation. Interestingly, even though the frequency and dissipation did not return to initial values, they did show a slight recovery after rinsing with PAO8, suggesting the presence of a partial desorption step, which was not present for the other POM-ILs. This was true for both substrates, but more substantially for 316L. Finally, SiW_9_THTDP showed a more consistent behavior between the two substrates. While Δ*f* dropped slightly more on 316L than on 52100, the difference was much smaller than for SiW_11_THTDP, suggesting a potentially weaker substrate dependence for this POM-IL. Furthermore, SiW_9_THTDP did not show the desorption step that was observed for SiW_11_THTDP, suggesting a strong chemisorption process.

Although the interpretation of the results for SiW_11_THTDA is more challenging given the dilution adjustments, the comparisons between SiW_11_THTDP and SiW_9_THTDP display a clear trend. This may indicate that the lacunarity of the POM, or the number of available cations, influences the affinity of the POM-ILs for the substrates.

### 3.3 Wear Analysis

The wear results have been divided into two sections: one for the compounds as additives and the second for them as plain substances.

#### 3.3.1 Wear Results of POM-ILs as Additives in PAO8

In Figure 5a, plain PAO8 showed the highest specific wear rate (SWR) for the 316L substrate. When additives were added to PAO8, wear reduced significantly to values around 4 × 10^–4^ mm^3^/Nm with the notable exception of SiW_9_THTDP, with values closer to 1.7 × 10^–4^, rendering this additive the most efficient at wear reduction on this substrate. For 316L, the beta (β) values for the POM-ILs had a small range, between 35 and 40%, showing a mainly plastic deformation of the substrate. ZDDP showed a higher value, closer to 60%, and plain PAO8 closer to 70%, which in both cases indicates a stronger contribution by abrasive wear.

**Figure 5:**
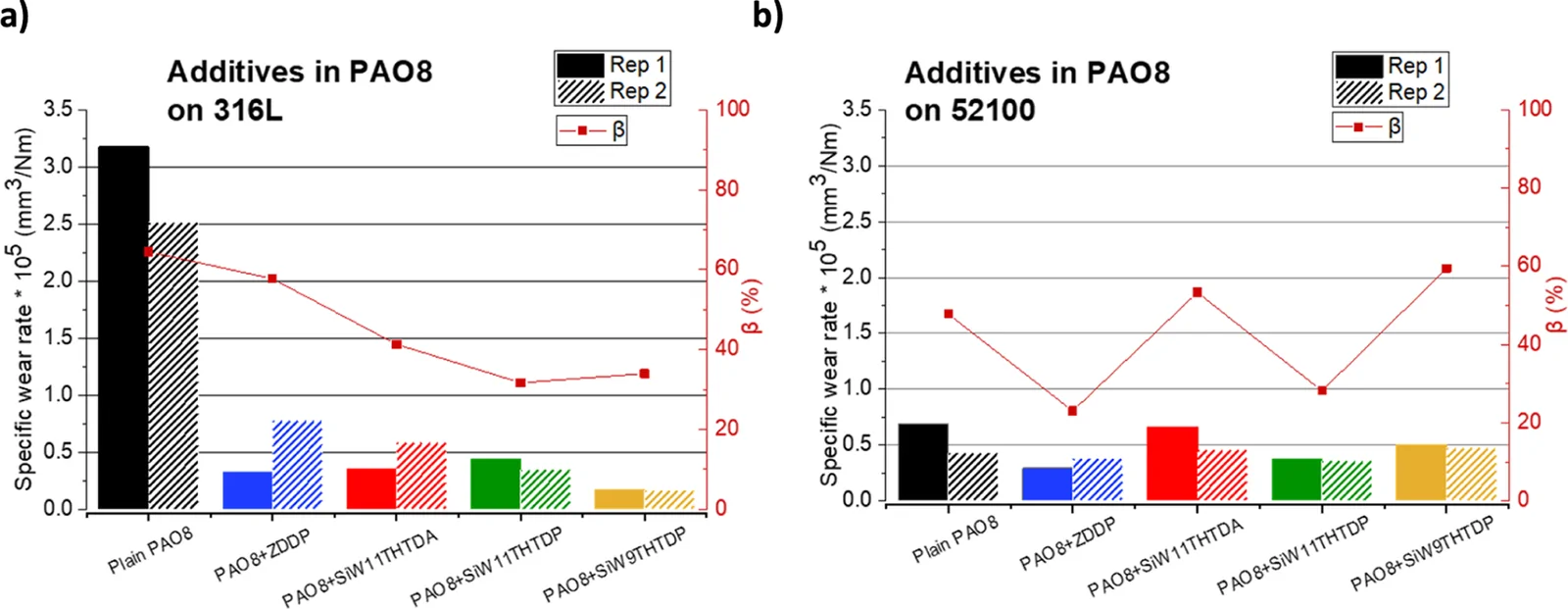
SWR and β value for POM-ILs and ZDDP as additives in PAO8 in 316L (a) and 52100 (b).

Figure 5b shows the results for the 52100 substrate. First, plain PAO8 shows very similar SWR to PAO8 with additives. Although the SWR are closer together for all additives, quite clear differences can be observed for the β values, where two groups are easily observed: Plain PAO8, PAO8-SiW_11_THTDA, and PAO8-SiW_9_THTDP with values between 50 and 60% and PAO8-ZDDP and PAO8-SiW_11_THDP, closer to 30%. This illustrates that the former group presents abrasive wear predominantly, whereas the latter shows more of a plastic deformation effect.

#### 3.3.2 Wear Results of Plain ZDDP and POM-ILs

While in 316L (Figure 6a), plain ZDDP and SiW_9_THTDP showed very low SWR, close to or below 0.1 × 10^–5^ mm^3^/Nm, SiW_11_THTDP showed a SWR increase of almost 10 times, displaying the different nature in the antiwear effectiveness of different POM-IL structures. The β values are also consistent between ZDDP and SIW_9_THTDP, of around 60%, while SiW_11_THTDP reaches 80%, all leaning into a predominant contribution from abrasive wear.

**Figure 6:**
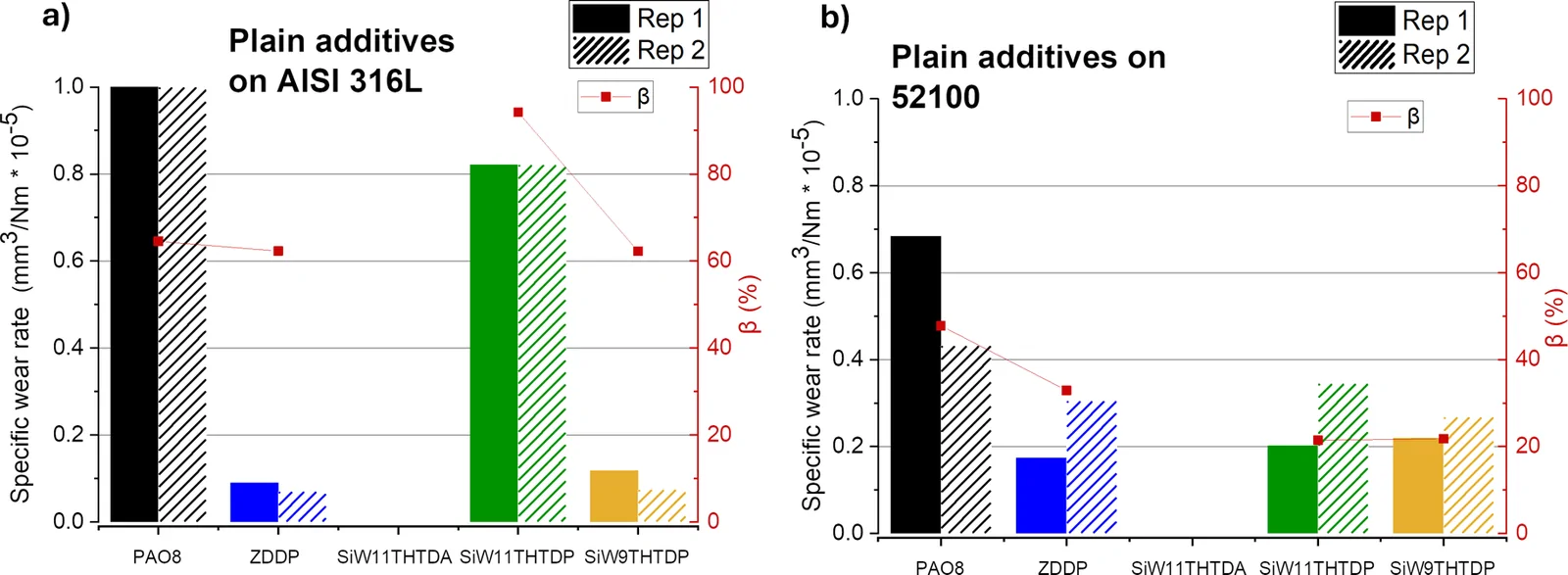
SWR and β value for two plain POM-ILs and ZDDP in 316L (a) and 52100 (b).

In the case of the 52100 substrate (Figure 6b), a much more homogeneous behavior of all species is observed. All lubricants present similar SWR and β values, with only a slightly larger β for ZDDP. This substrate therefore does not seem to discern the antiwear character of the different compounds.

### 3.4 Wear Morphology by SEM

#### 3.4.1 Wear Morphology of POM-ILs and ZDDP as Additives in PAO8

Figure 7 compares the topography of the wear tracks obtained after tribotesting with the different POM-ILs and ZDDP additives in PAO8 and plain PAO8.

**Figure 7:**
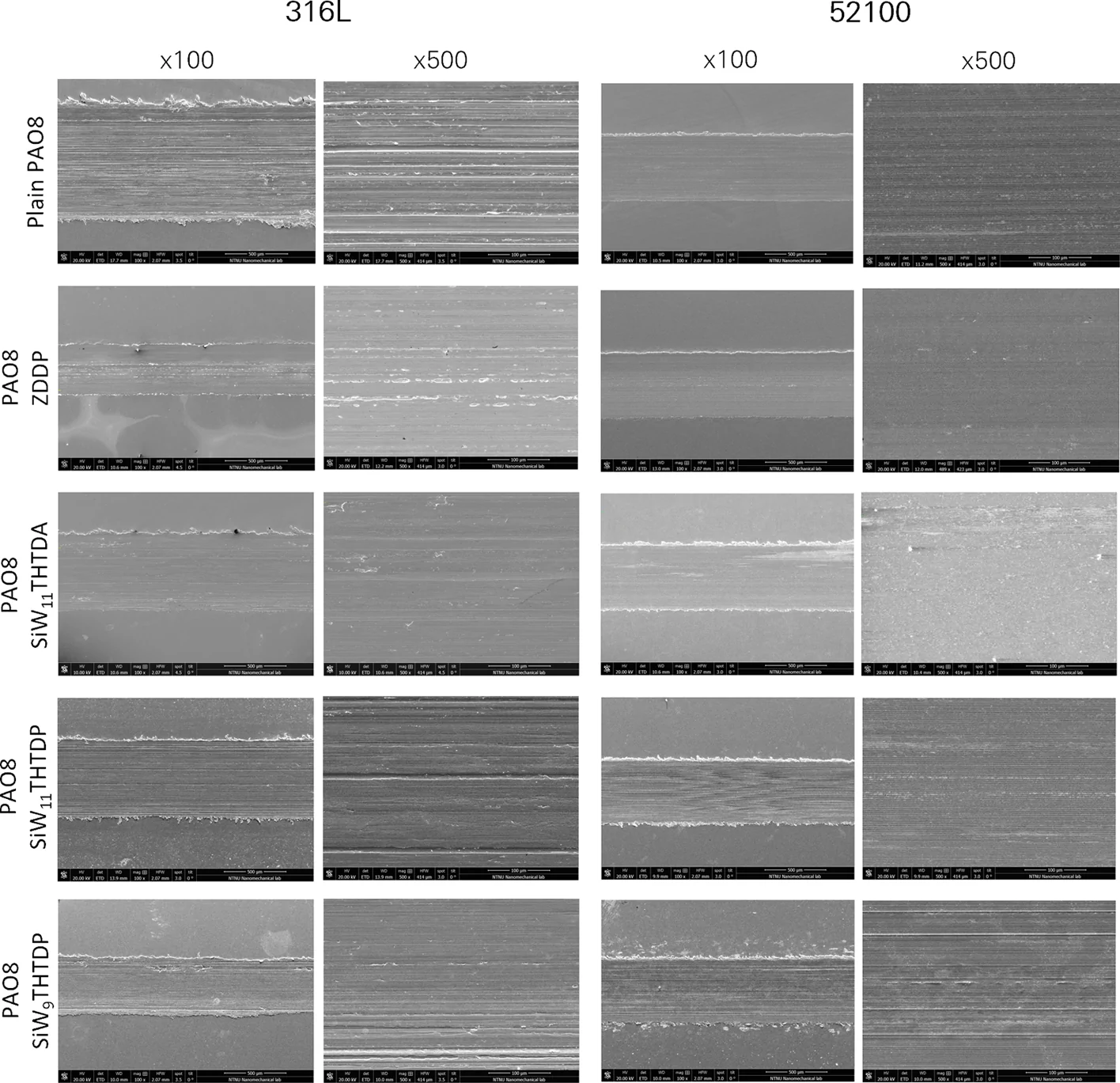
SEM top-view images of the wear tracks after testing with POM-ILs and ZDDP as additives in PAO8.

On 316L, plain PAO8 showed the highest degree of abrasive wear with deep, continuous grooves across the entire wear track that rendered the widest wear track of all tests. The addition of SiW_11_THTDP resulted in similarly deep grooves, but they were less uniformly distributed. The overall top view appeared less abraded than plain PAO8. Adding SiW_9_THTDP to PAO8 showed even lower abrasion, with wear marks concentrated along the edges of the scar while the central region remained relatively smooth. Among the POM-ILs, SiW_11_THTDA exhibited the smoothest surface with only shallow and sparse abrasion marks. ZDDP resulted in a similar degree of abrasion to SiW_11_THTDA and SiW_9_THTDP, but with its grooves concentrated in the center of the wear track and smoother regions near the edges. Notably, the grooves formed by ZDDP as an additive were more irregular, in contrast to the continuous lines observed for all of the other tests.

On 52100, all blends resulted in a lower degree of abrasion compared to 316L, consistent with the higher strength of 52100 as a material. Plain PAO8 on this substrate produced faint abrasive grooves, uniformly distributed throughout the wear scar. This pattern was also observed for both SiW_11_THTDP and SiW_9_THTDP, although the latter presented slightly less uniform grooves, and the deepest appeared close to the edges. Finally, both SiW_11_THTDA and ZDDP presented the lowest degree of visible abrasion inside the wear track in 52100. Although both additives presented a very smooth surface, ZDDP exhibited minor side ridges, while SiW_11_THTDA formed more pronounced edges along the wear track.

#### 3.4.2 Wear Morphology of Plain POM-ILs and ZDDP

In Figure 8, the plain POM-ILs and ZDDP are compared on the two tested substrates. Plain PAO8 is added for comparison.

**Figure 8:**
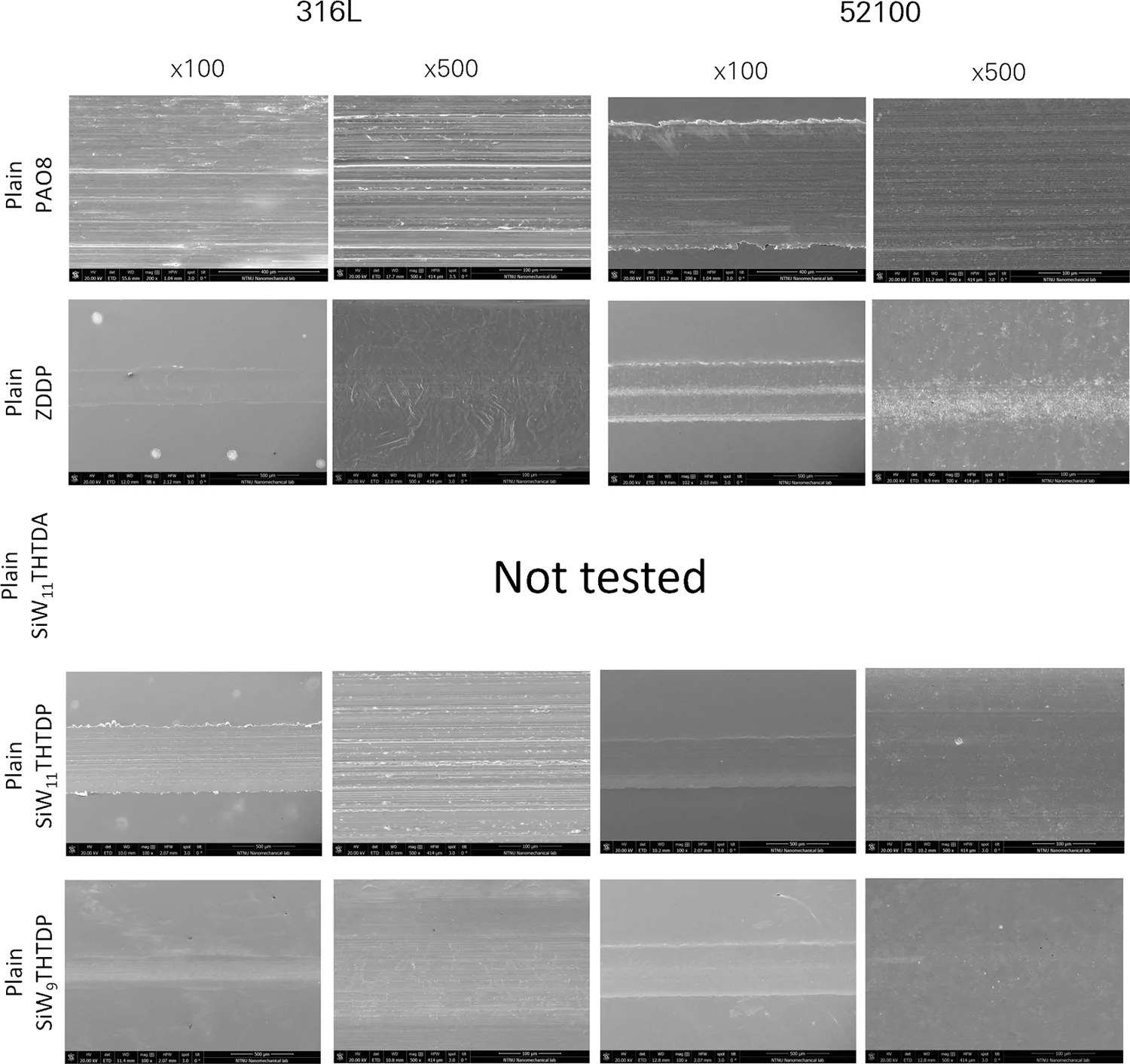
SEM top-view images of the wear tracks after testing with plain POM-ILs and ZDDP.

On 316L, plain SiW_11_THTDP produced deep, continuous abrasive grooves throughout the wear track, similar to that of plain PAO8. In contrast, SiW_9_THTDP exhibited an exceptionally faint wear track with poorly defined edges that made the wear scar boundaries hard to discern. Most wear marks were minimal and concentrated in the middle section of this wear track. A similar trend was observed for ZDDP, where a thin and faint wear scar was found. In this case, very minimal abrasive grooves are also present along with scattered scratches, possibly caused by loose wear debris.

On 52100, the surface topographies revealed different behavior when compared to 316L, with the notable exception of SiW_9_THTDP. Both plain PAO8 and SiW_11_THTDP exhibited a significant decrease in their degree of abrasion. In contrast, ZDDP resulted in the formation of a wider wear scar and the appearance of a more damaged surface where scattered pits, rather than grooves, were present throughout the central region, as well as the lower edge of the wear scar. SiW_9_THTDA presented minimal abrasion throughout the wear scar and a generally smooth track.

### 3.5 Wear Track Cross-Section Characterization by FIB SEM

The cross-sectional analysis of the wear tracks highlights clear trends in the subsurface deformation behavior induced in the metal substrates by the different lubricant blends and plain compounds. Plain PAO8 cross sections are added in both subsections (Figures 9 and 10) for comparison, showing extensive plastic deformation in 316L and limited plastic deformation in 52100.

**Figure 9:**
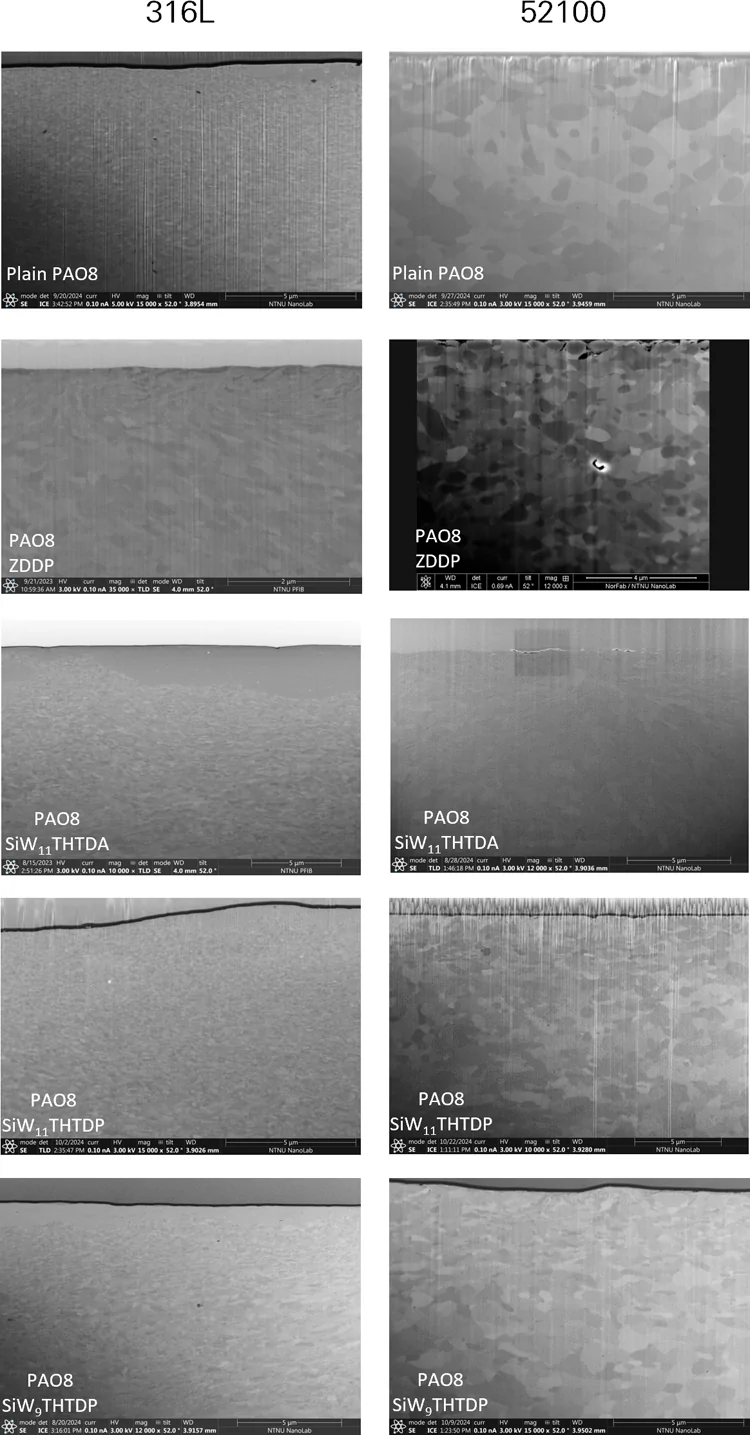
SEM-FIB cross-sectional images of 316L and 52100 after testing with POM-ILs and ZDDP as additives in PAO8.

**Figure 10:**
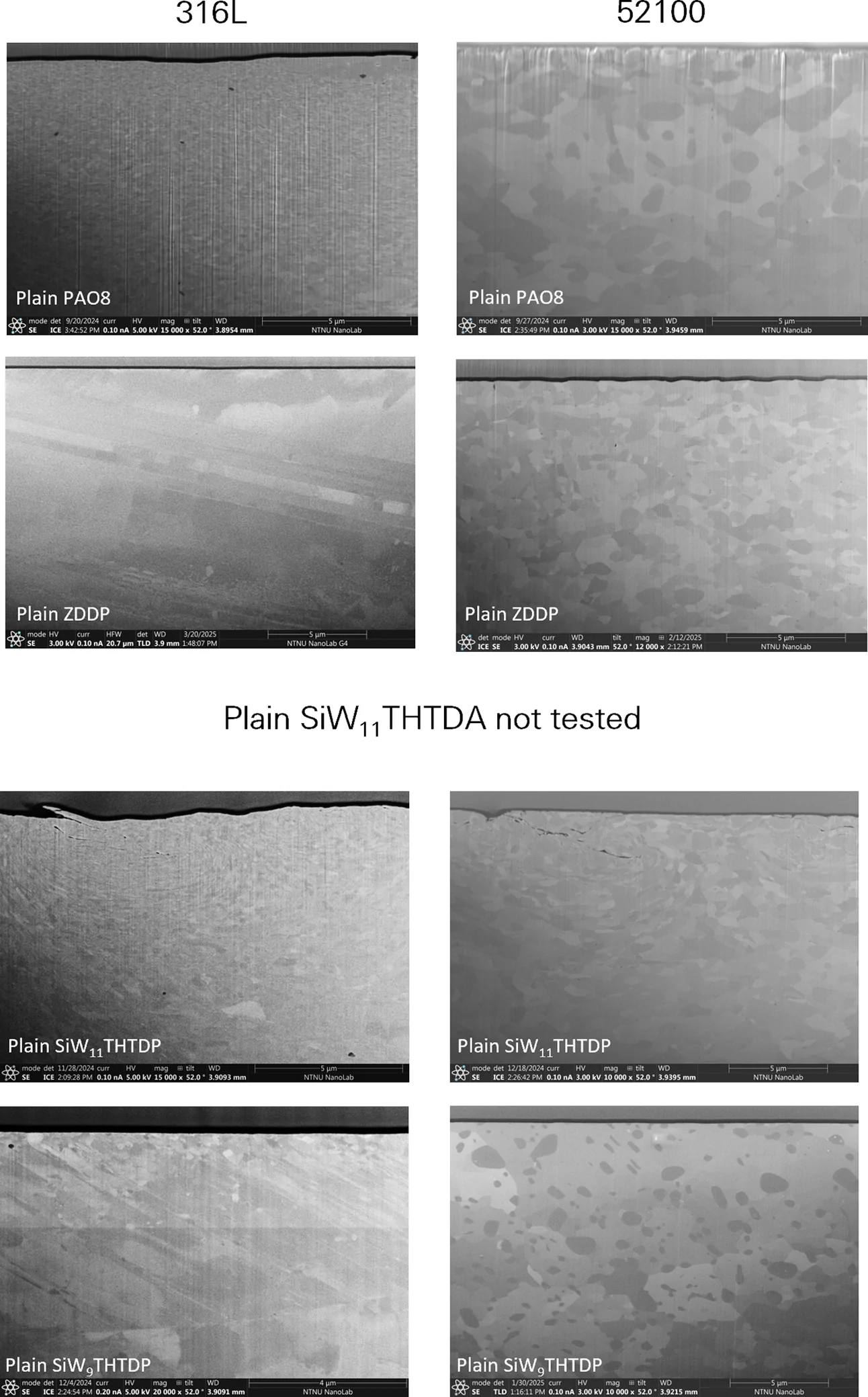
SEM-FIB cross-sectional images of 316L and 52100 after testing with plain POM-ILs and ZDDP.

#### 3.5.1 Cross-Sectional Images of POM-ILs and ZDDP as Additives in PAO8

On 316L (Figure 9), all POM-ILs generated a large recrystallization area, extending well over 10–15 μm into the substrate, indicating significant plastic deformation caused during sliding. Right beneath the surface, within the first few microns, an area of extreme grain refinement is observed, where the formation of nanosized grains is present. This effect is especially visible for SiW_11_THTDA and SiW_9_THTDP. The cross-section image of ZDDP showed grain elongation that rapidly transitions into the bulk of the material and no evidence of significant grain refinement. Additionally, the presence of a thin, irregular dark surface layer for ZDDP hints at the formation of a patchy tribofilm, as previously described in the literature.^15^

On 52100 (Figure 9), the transition from the deformed zone to the bulk of the material occurs at much shallower depths compared to 316L due to the higher strength and wear resistance of this material. On this substrate, the POM-ILs induced a slightly thicker region of elongated grains, although it was significantly less than in the case of 316L. ZDDP presented minimal plastic deformation and a rapid transition to the bulk structure.

#### 3.5.2 Cross-Sectional Images of Plain POM-ILs and ZDDP

When looking at the 316L substrate of Figure 10, it is interesting to note that SiW_11_THTDP produced a similar microstructure to when it was used as an additive, presenting a significant region of grain refinement and plastic deformation that transitions back to the bulk structure after around 5 μm. In contrast, SiW_9_THTDP showed evidence of minimal recrystallization of a few hundred nanometers before restoring to the bulk microstructure of 316L. ZDDP shows similar behavior to SiW_9_THTDP.

On 52100, clear differences in the subsurface microstructure were also observed between the different compounds. For the POM-ILs and ZDDP, the trends mostly mirrored those seen for 316L, although in this case at shallower depths. SiW_11_THTDP exhibited the largest degree of grain refinement and elongation. In contrast, both ZDDP and SiW_9_THTDP, the latter to a larger degree, showed minimal effects on the subsurface microstructure, with a very shallow region of plastic deformation.

### 3.6 Surface Chemical Analysis by XPS

X-ray photoelectron spectroscopic analyses were carried out inside and outside the wear track on both metal substrates for ZDDP, SiW_11_THTDA, SiW_11_THTDP, and SiW_9_THTDP used as additives in PAO8. Each sample underwent a survey scan, providing a wide range of binding energy values to identify elements on the substrate’s surface, with lower resolution and sensitivity. High-resolution scans were also performed, focusing on narrower energy windows to deliver more sensitive and detailed elemental analysis. While survey scans provide elemental quantification based on atomic concentration distribution, they often lack the resolution necessary to detect small traces of elements and may result in over- or underestimations. On the other hand, high-resolution scans allow identification of differences in the chemical environment of elements and provide more reliable results of elemental presence. Additionally, etching was conducted to understand the elemental evolution within each additive. However, given the time-consuming nature of these analyses, etching was only performed on key samples.

The high-resolution scans for additives on PAO8 on both 316L and 52100 are presented in Figure 11, where the different elements are assigned according to reported peaks for S, Zn, P, and W.^16^ ZDDP clearly shows the presence of key elements (S, Zn, and P) inside the wear tracks of both metals to similar extents (Figure 11), indicating no significant substrate dependence for this additive. The POM-ILs, however, displayed notable substrate-dependent behavior. Strong W signals were observed inside the wear track for 316L, whereas they were much less intense for 52100. As etching proceeded, the high-resolution scans also revealed the presence of a secondary oxidation state for W, particularly for SiW_11_THTDA and SiW_11_THTDP, and to a lesser extent for SiW_9_THTDP. Interestingly, SiW_9_THTDP appeared to induce a less intense W signal in 316L compared with the other two POM-ILs. It is important to note that although binding energy intensities alone should not be used for definitive elemental quantification, they can provide valuable indications.

**Figure 11:**
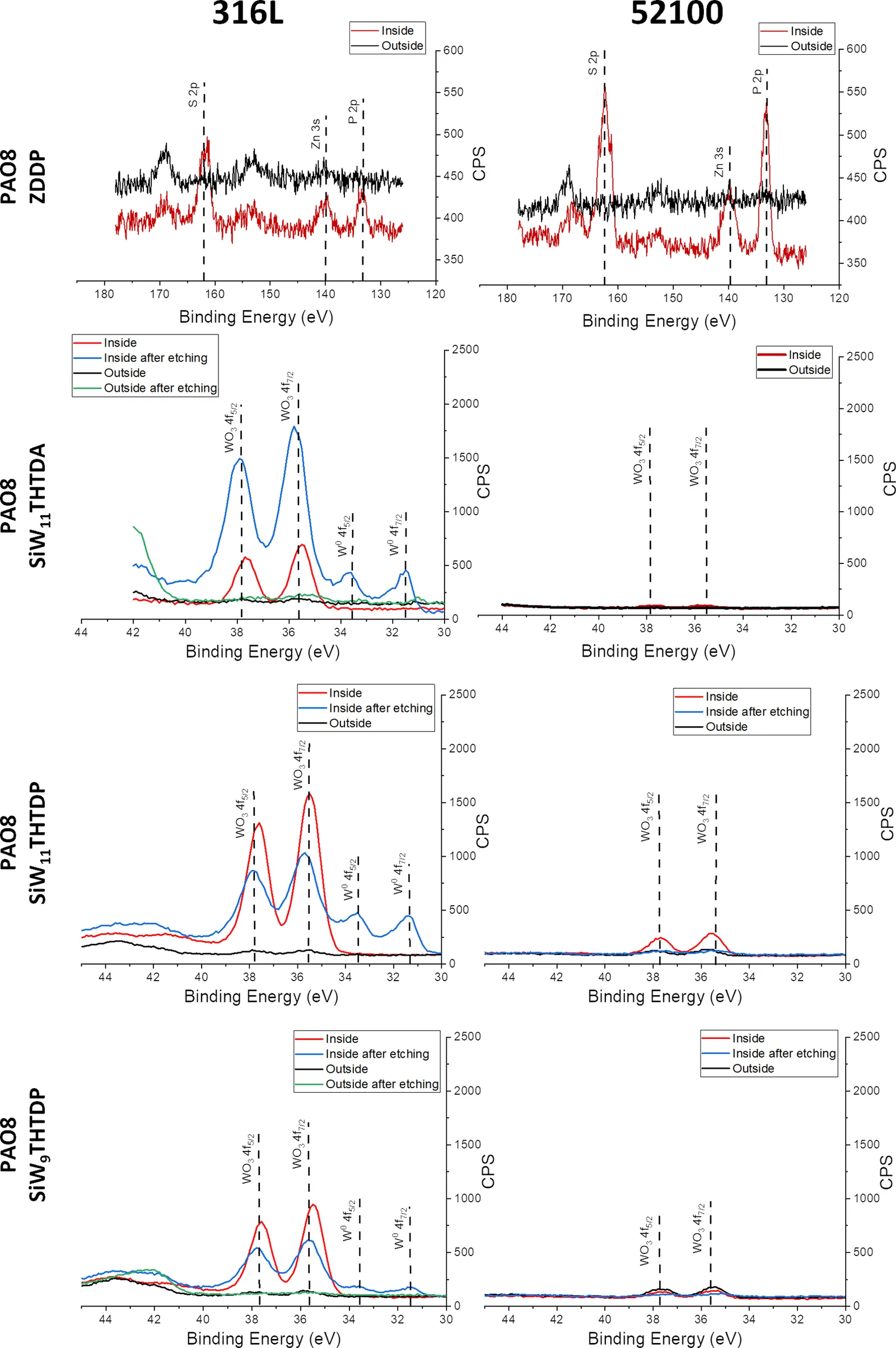
High-resolution XPS spectra of the metal substrates inside and outside the wear track for all additives in PAO8. For some samples, spectra obtained after an etching process are also included.

A similar set of experiments were carried out on the metal substrates tested with plain ZDDP, SiW_11_THTDP, and SiW_9_THTDP (Figure 12). In this set of analyses, ZDDP showed a minimal presence of P or S atoms on both substrates. Nonetheless, traces of P were observed on 52100 to a similar extent inside and outside the wear track; however, etching removed this contribution. Interestingly, SiW_11_THTDP displayed strong W signals inside the wear tracks of both metals, with especially intense peaks on 316L. As with the POM-ILs as additives, the W peaks for SiW_11_THTDP evolved into a different oxidation state as the etching process proceeded on 316L. Notably, the resulting W peaks for SiW_9_THTDP were weak, with no evidence of oxidation-state changes observed for 52100. A comparable pattern was observed for 316L, which contrasts with the general POM-IL trend of strong W detection on this substrate.

**Figure 12:**
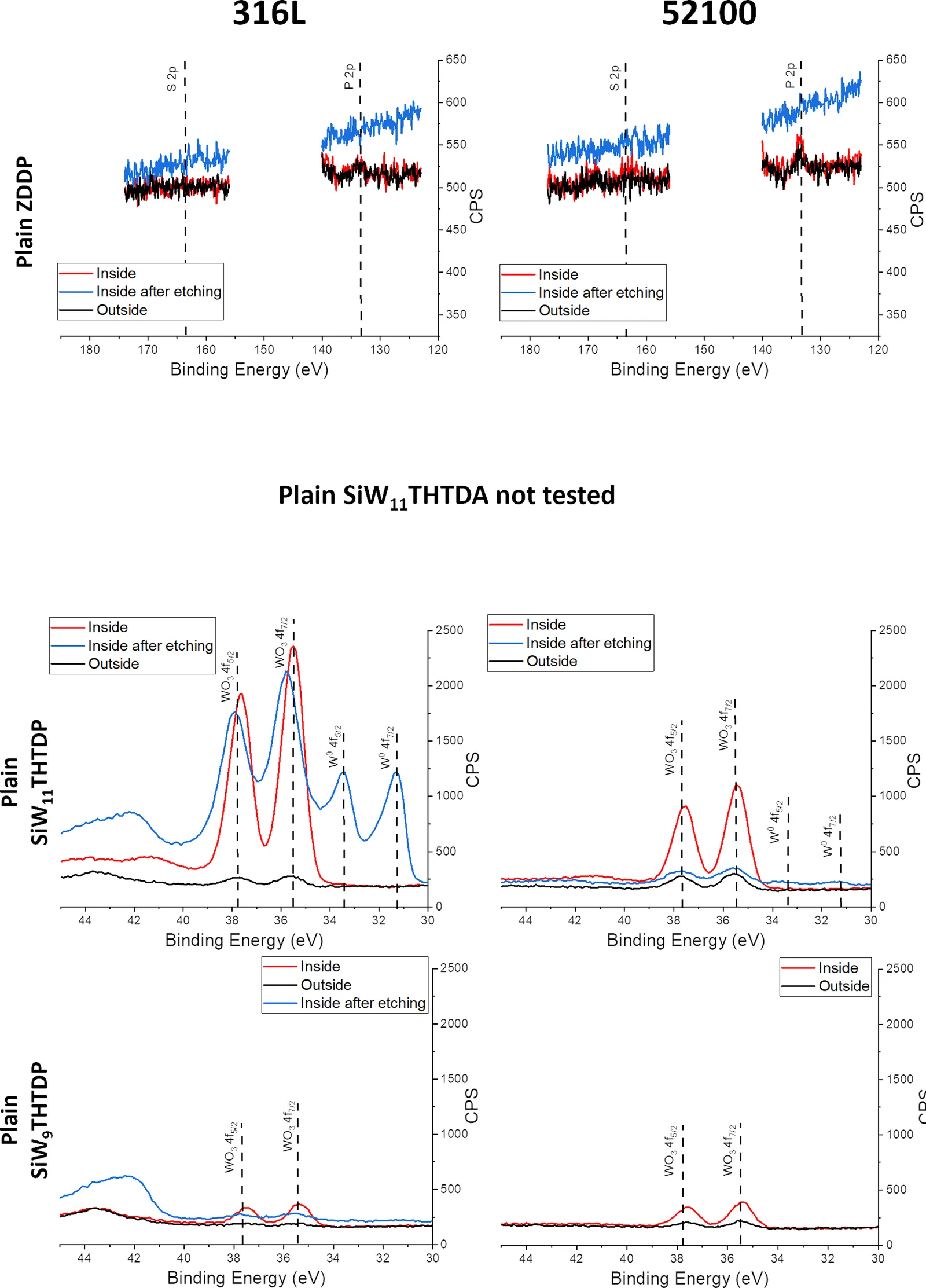
High-resolution XPS spectra of the metal substrates inside and outside the wear track for all plain compounds. For some samples, spectra obtained after an etching process are also included.

## 4 Discussion

### 4.1 Effect of the Cation Moiety on Friction and Wear of POM-IL Additives in PAO8

The aim behind testing different POM-IL structures was to find a structure-to-tribological performance trend or relationship for this family of compounds. Even though a generalization is out of reach for this study, given the limited number of modifications studied, some correlations can be observed. The first POM-IL modification was swapping the nature of the central atom in the cationic moiety, shifting from an ammonium (THTDA)- to a phosphonium (THTDP)-based cation. This was strategized to bring the POM-IL closer to environmental consciousness, given the potential toxic properties of ammonium-based ILs, compared to phosphonium.^17–19^

This first modification did not induce any notable effects on either the friction or wear reduction of SiW_11_THTDP compared with SiW_11_THTDA when used as additives in PAO8. Just a slightly shorter running-in period on 316L and a smaller wear rate on 52100 were observed for SiW_11_THTDP. However, SiW_11_THTDA seemed to present a smoother wear track, which might stem from experimental deviation alone. This suggests therefore that only changing the nature of the atom in the cation moiety of the POM-IL has a negligible impact on its tribological performance when used as an additive in PAO8. Although a cation containing phosphorus (a triboactive element) can suggest an improvement in tribological response, this was not observed in this study. Thus, these results further emphasize the hypothesis of the little influence of the cation in the tribological performance of ionic liquids [8].

### 4.2 Effect of the Anion Moiety on Friction and Wear of POM-IL Additives in PAO8

Changing the anion did come with more significant variations in tribological behavior, especially on wear. This is to be expected given the results from previous works showing a stronger contribution of the anionic moiety of ILs in tribological systems.^8,20^ For studying this effect, the SiW_11_THTDP and SiW_9_THTDP POM-ILs were used. Figure 13 shows a summary of the friction and wear results for these compounds both in PAO8 and plain.

**Figure 13:**
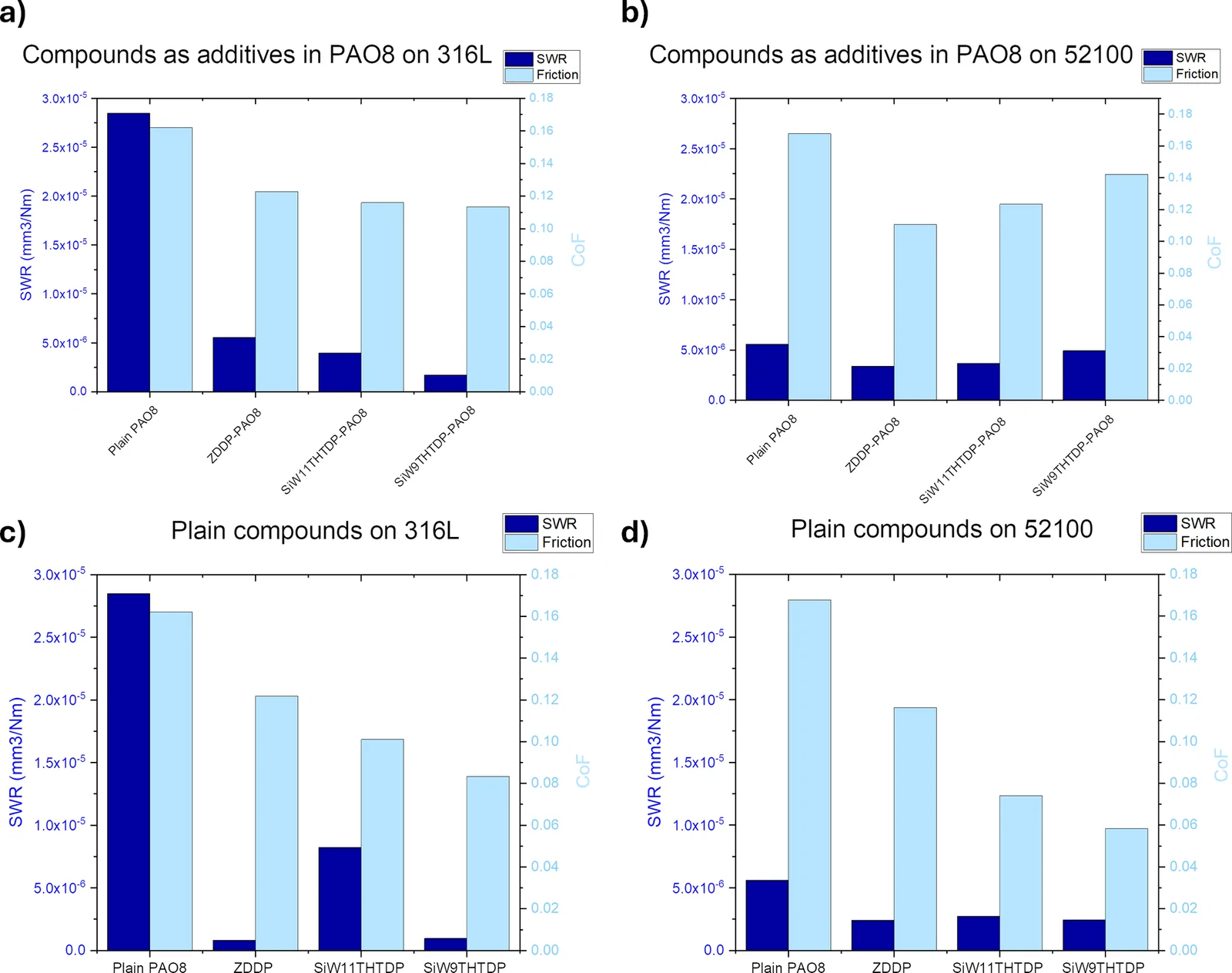
Friction and wear comparison for ZDDP, SiW_11_THTDP, and SiW_9_THTDP as additives in PAO8 on 316L (a), 52100 (b), and plain lubricants on 316L (c) and 52100 (d).

Friction differences were minimal when using SiW_11_ or SiW_9_ anions for both substrates as additives in PAO8, and the differences in wear were most significant on 316L both when using them as additives and plain. On 52100, PAO8-SiW_11_THTDP resulted in a mildly lower wear rate than PAO8-SiW_9_THTDP, and it was virtually the same when used as plain compounds.

On 316L, the wear rate for PAO8-SiW_11_THTDP is higher than that for PAO8-SiW_9_THTDP, which is further corroborated by the SEM images (Figure 7), where a clearly narrower wear track is observed for PAO8-SiW_9_THTDP. This strongly suggests that anion modification has an impact on the antiwear performance of POM-ILs on 316L. Indeed, the results when testing the plain compounds support that effect (Figure 13), where a drastic reduction (eight times lower) in wear is found for SiW_9_ vs SiW_11_. The shift from SiW_11_ to SiW_9_ came with the removal of 2 atoms of tungsten, which in turn increased the lacunary character of the POM, opening up the structure and thus increasing the accessibility of terminal oxo ligands^21^ to the metal substrate. Furthermore, the overall net negative charge of the anion changed from 8 to 10 (Table 1) with its subsequent increase in countercations, making SiW_9_THTDP an overall bulkier and more electronegative POM-IL. Additionally, an important parameter to consider in terms of both redox action and overall stability is the q/m ratio,^22^ which also increases moving from SiW_11_ to SiW_9_.

One possible explanation could be that the higher negative charge on the POM anion would increase its affinity for the positively charged metal surface generated during sliding, thereby enhancing the kinetics and effectiveness of protective layer formation. However, in a previous study^10^ on the PAO8-SiW_11_THTDA system, we concluded that strong substrate affinity alone did not suffice for achieving antiwear and friction-reducing behavior in the case of POM-ILs. This is further supported by the present study’s adsorption results for PAO8-SiW_11_THTDP and PAO8-SiW_9_THTDP, particularly the case for 316L (Figure 4), where at equal concentrations, SiW_11_THTDP induces a significantly larger frequency drop, strongly suggesting greater substrate affinity. Therefore, this hypothesis can be ruled out, as it fails to explain why an additive with lower substrate affinity (in terms of adsorption) leads to improved antiwear performance.

Another possible explanation for the observed behavior is the formation of a viscoelastic, dampening layer with efficient antiwear properties. This could be facilitated, especially in the case of SiW_9_THTDP, as its ten surrounding cations may promote the formation of micelle-like structures that act as physical barriers between the surfaces. In this scenario, the cations would play an active role facilitating the separation of the sliding surfaces, thus leading to significantly lower wear. Although their presence increases the stearic hindrance for the POM to reach the metal substrate (Figure 4), they may contribute to optimal viscoelasticity and dampening, thereby reducing wear and shortening the running-in period. Recent literature supports this interpretation, suggesting that POM-ILs exhibit bulk, supramolecular ordering, and collective motion rather than behaving as discrete molecular units. This dynamic, bulk organization could justify POM-ILs’ macroscopic behavior of viscoelastic layer formation, which otherwise might clash with their expected behavior as independent molecules.^23^

The increased cationic presence for SiW_9_THTDP may hinder or delay the access of the POM to the metal surface compared to SiW_11_THTDP. This is supported by the weaker XPS W peak intensities as well as the at. % ratio between Fe and W observed on the discs lubricated for PAO8-SiW_9_THTDP. Toward the end of the etching process, this ratio remains between 14 and 16 for SiW_11_THTDP but rises to 90 for SiW_9_THTDP (Figure S3), indicating a significantly higher Fe contribution relative to W for the latter. Therefore, increased W presence on the wear track does not seem to be the sole reason for improved antiwear or reduced friction (Figure 13), and thus other processes might be at play.

Moreover, as shown in our previous work,^7^ as the tribotests progress and local temperatures exceed 200 °C, the cationic moiety of the POM-IL begins to decompose, allowing increased access of the POM moiety to the surface. This can lead to the formation of W deposits, as observed in Figure 11, which is strong evidence that the POM reacts with the 316L surface, leading to hardening and grain refinement of 316L, which in turn enhances its wear resistance. This suggests that there might be a combined effect of chemical interaction with the metallic surface, which hardens the substrate and induces W–Cr interaction, measured on XPS, and physical separation of the surfaces by a viscoelastic film, rich in “POM-IL micelles”, which seemingly make SiW_9_THTDP particularly effective in minimizing wear.

### 4.3 Role of the Base Oil (PAO8) on the Tribological Performance of POM-ILs

Figure 13 visually summarizes the friction and wear behavior of the plain POM-ILs and the PAO8-POM-ILs blends on both substrates. Notably, both plain POM-ILs exhibited improved friction-reducing behavior on 52100 compared to ZDDP, in contrast to their poorer behavior when blended in PAO8. However, the wear rate remained relatively similar for the three lubricants on this substrate. These observations suggest that the presence of PAO8 as a lubricant base may be detrimental for POM-ILs on 52100. In the case of 316L, the effects were stronger on wear rather than friction and were most significant for the plain compounds, again confirming a detrimental effect of PAO8 on their performance.

Friction and wear can be categorized as physically and chemically driven processes, respectively. Previous experimental results,^7,10^ corroborated in the current work (Figures 11 and 13), showed significant wear reduction on 316L followed by the presence of a strong W signal on the wear track surface and near-surface. This observation was accompanied by the evidence of redox processes and Cr–W interactions, alongside hardening and grain refinement.^10^ This suggests that, at least for 316L, the antiwear performance of POM-ILs goes through a clear tribochemical process.

On the other hand, the observation that both plain SiW_11_THTDP and SiW_9_THTDP significantly reduced friction but did not have any effect on wear for 52100 might support the physically driven process for friction. The XPS results for plain POM-ILs showed minimal W presence on 52100 (Figure 12) and a stronger chemical affinity on 316L. Indeed, the chemical affinity of POM-ILs seems stronger on 316L than on 52100, as evidenced by the XPS and QCM results, which is always accompanied by a stronger effect on wear reduction for 316L. Thus, for 52100, a more efficient physical separation of the surfaces, the role of lubricant viscosity, or the inherent wear resistance of 52100 may play a more significant role in friction evolution. When the POM-IL was blended in PAO8 and tested on 52100, the POM-IL clusters might be suspended in the base fluid, and their interaction with the surface, mostly electrostatic, is suppressed by the presence of long, nonpolar base fluid molecules, rendering the system more static. The absence of PAO8 strengthened the POM-ILs affinity for the substrate, allowing the POM-IL clusters, or “POM-IL micelles”, to reach the metal surface more effectively, promoting friction reduction through physical separation and dampening, an effect not observed for ZDDP, which exhibited almost twice the friction.

The structural differences between the two POM-ILs can explain their friction performance variations. As shown in previous results (Figures S1, S5, S7 and S11), SiW_9_THTDP appears especially efficient in forming micelle-like structures and thus a better dampening layer (Figure 14), supporting its enhanced friction-reducing character (Figure S13). Even though the effectiveness of the SiW_11_THTDP micelle-like structures is slightly reduced because of the lower number of cationic moieties, it is still sufficient to lower the friction values with respect to ZDDP. Furthermore, since the W–Cr bonding does not apply to 52100, the wear reduction observed for plain POM-ILs likely stems from improved shear efficiency and surface separation, rather than chemical reactions.

**Figure 14:**
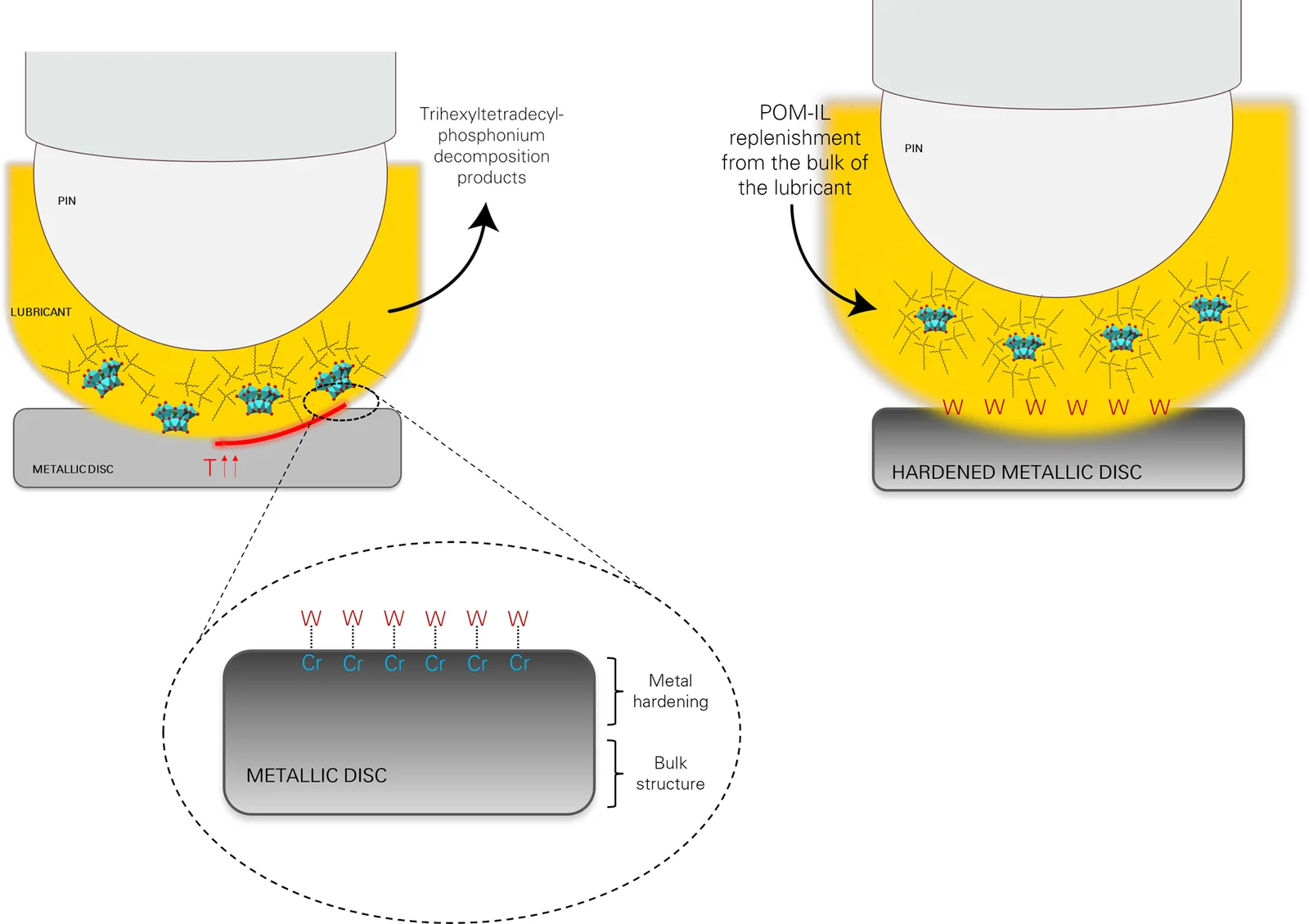
Proposed friction-reducing and antiwear mechanism of SiW_9_THTDP in PAO8 on 316L.

These findings support the hypothesis that POM-ILs, to different extents depending on their three-dimensional structure, act as multifunctional additives in a sequential manner: antiwear agents (upon their chemical interaction with, especially, the 316L surface^10^ that seems to harden and protect the surface) and friction reducers (through the presence of the protective dampening film, formed of “POM-IL micelles” that separate the surfaces), as can be observed in Figure 14, with SiW_9_THTDP as an example.

To test the proposed hypothesis, we carried out a series of tests at a higher normal load (40 N) as an attempt to induce failure of the viscoelastic film, which would in theory provide much more significant friction and wear, especially for SiW_9_THTDP. The friction and wear results for these experiments are found in the Supporting Information, Figures S1 and S2. These results show a very similar trend to that observed for the experiments at 20 N, where the running in during the friction evolution was shorter for SiW_9_THTDP than SiW_11_THTDP. Additionally, the wear rate was much lower for SiW_9_THTDP, just as was the case for the 20 N experiments. Consequently, it is likely that the viscoelastic film formed is very resistant, and higher loads would be needed to induce its failure.

## 5 Conclusions

The results presented in this work demonstrate that the molecular design of POM-ILs significantly influences their tribological performance, establishing a clear relationship between the structure and function for this class of multifunctional lubricant additives. Both cationic and anionic modifications were introduced and tested on two metallic substrates, AISI 316L stainless steel and annealed AISI 52100 bearing steel, under boundary lubrication conditions. The compounds were evaluated as both plain lubricants and additives in PAO8, and tribological testing was combined with adsorption, surface, and subsurface characterization to evaluate the mechanisms governing their tribological performance.

The results demonstrated that cationic modification (THTDA → THTDP) exhibited negligible effect on friction or wear, suggesting that the nature of the central atom in the cation plays a minor role in the overall tribological behavior of POM-ILs under the studied conditions. In contrast, anionic modification (SiW_11_ → SiW_9_) significantly influenced performance for POM-ILs used both plainly and as additives. Although the increased lacunarity and negative charge density of the SiW_9_ structure reduced both friction and wear on 316L, it resulted in lower surface affinity and tribochemical activity compared to SiW_11_, suggesting the influence of other factors in determining tribological performance.

The presence of PAO8 as a base lubricant decreased the tribological benefits of the POM-ILs relative to their plain form, suggesting that the base lubricant molecules inhibit the direct interaction of the POM-ILs with the substrate and thus reduce their ability to adsorb, organize, or undergo tribochemical reactions at the surface.

From the combined friction, wear, adsorption, and surface analyses, a two-step action mechanism is proposed for POM-ILs:

1. A chemically driven antiwear mechanism, where tungsten-containing POM moieties interact with the Cr-rich surface of 316L, promotes tribochemical reactions and substrate hardening.
2. A physically driven friction-reducing mechanism in which micelle-like POM-IL aggregates form viscoelastic, protective films that facilitate surface separation and wear reduction.

Overall, these findings confirm that the molecular design of POM-ILs can influence tribochemistry and substrate mechanical modification. While ZDDP remains a widely used antiwear additive, its environmental and mechanical limitations emphasize the potential of POM-ILs as environmentally acceptable, tunable, multifunctional alternatives. The insights obtained in this work highlight the role of rational molecular design and provide direction for optimizing POM-ILs in next-generation lubrication systems.

## Supplementary Material



## Notes

Average coefficient of friction values and repeatability data for all lubricant systems, supplementary friction and wear results obtained under a 40 N applied load, and XPS depth-profile and atomic concentration analyses used to evaluate tungsten distribution and tribochemical interactions on the tested metallic substrates (PDF)
