# Supplementary material for: Molecular Structure–Tribological Performance Relationship in Tungsten-Based Polyoxometalate Ionic Liquid Lubricant Additives
Source: ACS Omega. 2026 Aug 13;11(33):48894–911. doi: 10.1021/acsomega.5c12084 (PMC13520010; doi:10.1021/acsomega.5c12084)
Supplement: Supplementary file 1 [file ao5c12084_si_001.pdf]

Supplementary material

Molecular Structure–Tribological Performance Relationship in Tungsten-Based Polyoxometalate Ionic Liquid Lubricant Additives

María Luisa Casasin-Garcia[1], Scott Mitchell[2], Nuria Espallargas[1]

[1] Norwegian Tribology Center, Department of Mechanical and Industrial Engineering, Norwegian University of Science and Technology (NTNU), Trondheim, Norway.
[2] Instituto de Nanociencia y Materiales de Aragón (INMA-CSIC), Consejo Superior de Investigaciones Científicas, Universidad de Zaragoza, Zaragoza, Spain.

Supplementary material

Table S1. Averaged friction values for both repetitions on 316L stainless steel and 52100 bearing steel.

**Average COF after running in 316L rep 1**				
SiW11THTDA	SiW11THTDP	SiW9THTDP	ZDDP	PAO8
0.119390582	0.155090137	0.106510389	0.109770915	0.18448115

**Average COF after running in 316L rep 2**				
SiW11THTDA	SiW11THTDP	SiW9THTDP	ZDDP	PAO8
0.121819049	0.116012039	0.113379399	0.122811209	0.16214726

**Average COF 52100 rep 1**				
SiW11THTDA	SiW11THTDP	SiW9THTDP	ZDDP	PAO8
0.147329436	0.123340197	0.142143273	0.110683134	0.167739399

**Average COF 52100 rep 2**				
SiW11THTDA	SiW11THTDP	SiW9THTDP	ZDDP	PAO8
0.14462112	0.135537978	0.149851158	0.114349077	0.14565023

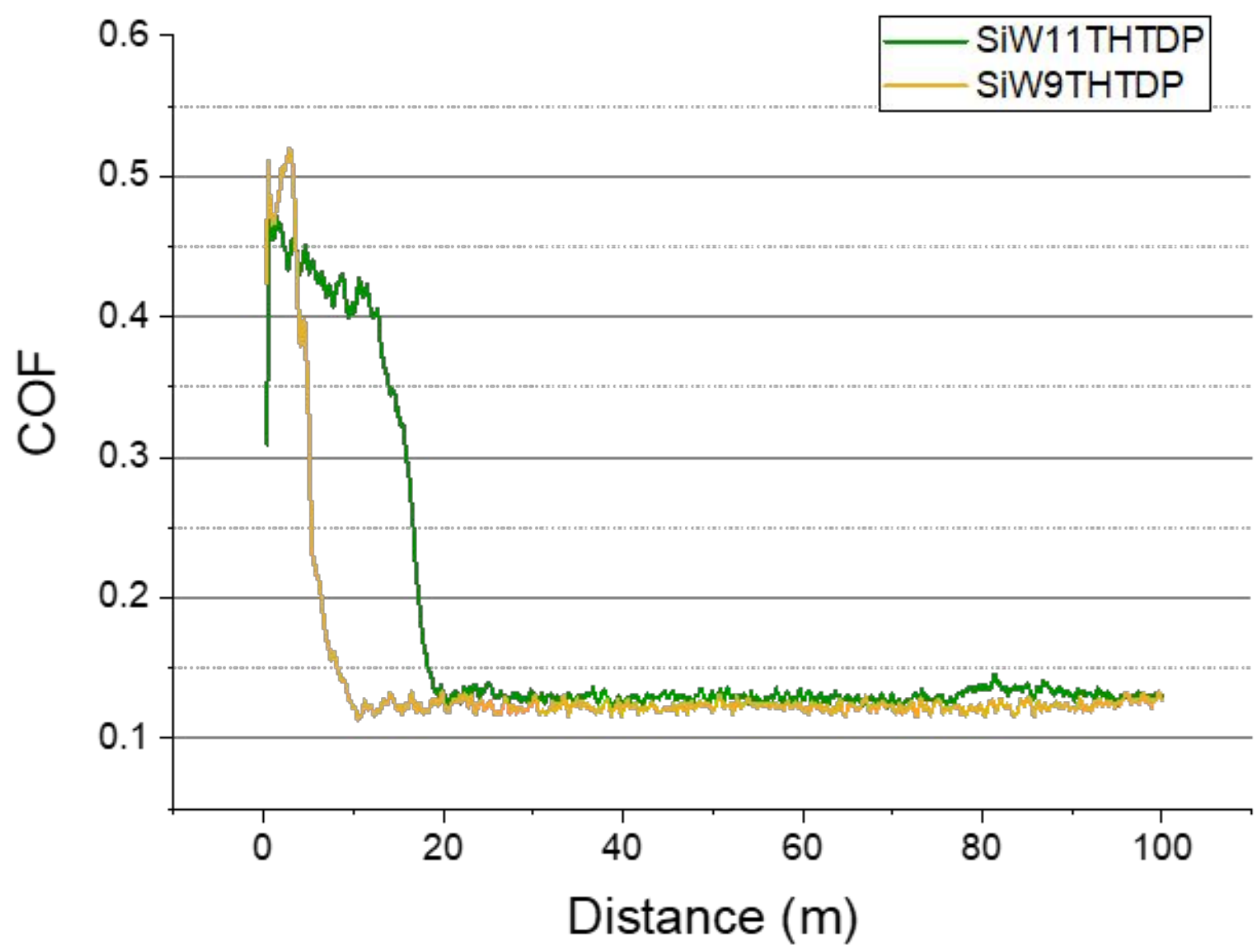

Figure S1. Friction evolution of two POM-ILs in PAO8 at a higher load of 40 N.

Supplementary material

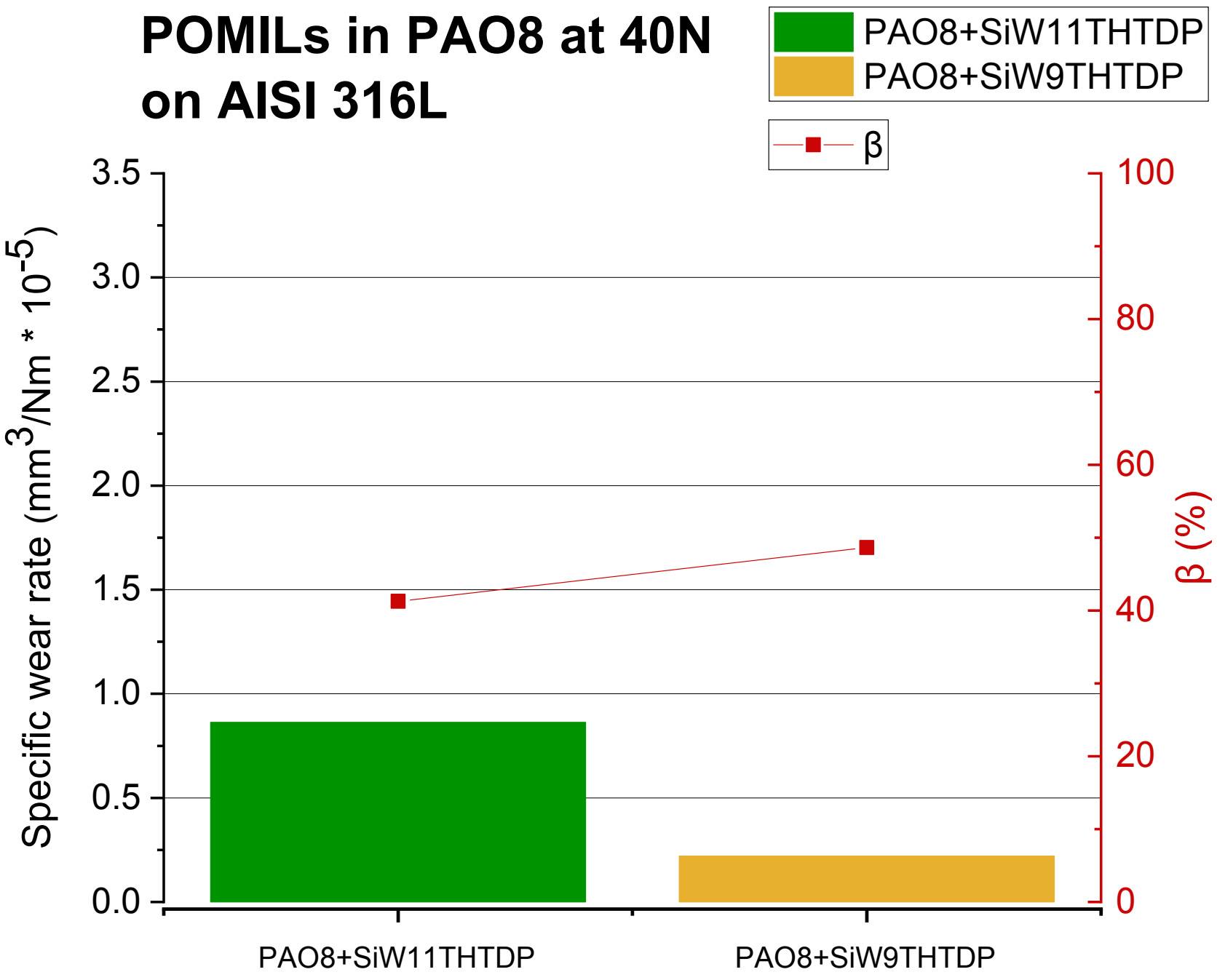

Figure S2. SWR and β value for two POM-ILs in PAO8 at a higher load of 40 N on 316L.

SiW11THTDP

Name	At%
O 1s	33.61
C 1s	5.47
Ni 2p	13.93
Fe 2p	35.27
W 4d	2.55
Cr 2p	7.99
Mo 3d	1.17

SiW9THTDP

Name	At%
O 1s	25.37
C 1s	24.09
Ni 2p	11.41
Fe 2p	29.12
Cr 2p	8.35
Mo 3d	1.33
W 4d	0.32

Figure S3. Casa XPS survey scan atomic distribution results for plain SiW_{11}THTDP and SiW_9THTDP.
